# Fitted filtration efficiency and breathability of 2-ply cotton masks: Identification of cotton consumer categories acceptable for home-made cloth mask construction

**DOI:** 10.1371/journal.pone.0264090

**Published:** 2022-03-22

**Authors:** Ken G. Drouillard, Amanda Tomkins, Sharon Lackie, Scott Laengert, Allison Baker, Catherine M. Clase, Charles F. De Lannoy, Dora Cavallo-Medved, Lisa A. Porter, Rebecca S. Rudman

**Affiliations:** 1 Great Lakes Institute for Environmental Research, University of Windsor, Windsor, ON, Canada; 2 School of Biomedical Engineering, McMaster University, Hamilton, ON, Canada; 3 Department of Chemical Engineering, McMaster University, Hamilton, ON, Canada; 4 Department of Biomedical Sciences, University of Windsor, Windsor, ON, Canada; 5 Department of Medicine, McMaster University, Hamilton, ON, Canada; 6 Windsor Essex Sewing Force, Community Volunteer Group, Windsor, ON, Canada; Universiti Teknologi Malaysia - Main Campus Skudai: Universiti Teknologi Malaysia, MALAYSIA

## Abstract

The objective of this study was to characterize commercially-available cotton fabrics to determine their suitability as materials for construction of cloth masks for personal and public use to reduce infectious disease spread. The study focused on cottons because of their widespread availability, moderate performance and they are recommended for inclusion in home-made masks by international health authorities. Fifty-two cottons were analyzed by electron microscopy to determine fabric characteristics and fabric weights. Sixteen fabrics were selected to test for breathability and to construct 2-ply cotton masks of a standard design to use in quantitative fit testing on a human participant. Cotton mask fitted filtration efficiencies (FFEs) for 0.02–1 μm ambient and aerosolized sodium chloride particles ranged from 40 to 66% compared with the mean medical mask FFE of 55±2%. Pressure differentials across 2-ply materials ranged from 0.57 to > 12 mm H_2_O/cm^2^ on samples of equal surface area with 6 of 16 materials exceeding the recommended medical mask limit. Models were calibrated to predict 2-ply cotton mask FFEs and differential pressures for each fabric based on pore characteristics and fabric weight. Models indicated cotton fabrics from 6 of 9 consumer categories can produce cloth masks with adequate breathability and FFEs equivalent to a medical mask: T-shirt, fashion fabric, mass-market quilting cotton, home décor fabric, bed sheets and high-quality quilting cotton. Masks from one cloth mask and the medical mask were re-tested with a mask fitter to distinguish filtration from leakage. The fabric and medical masks had 3.7% and 41.8% leakage, respectively. These results indicate a well fitted 2-ply cotton mask with overhead ties can perform similarly to a disposable 3-ply medical mask on ear loops due primarily to the superior fit of the cloth mask which compensates for its lower material filtration efficiency.

## Introduction

The COVID-19 global pandemic placed unprecedented demands on global stocks of personal protective equipment (PPE) such as N95 respirators and certified medical masks [1, 2]. Owing to the rapid surge in PPE demand early in the pandemic, there were grave fears about shortages of PPE for frontline health care workers especially given that most commercial PPE recommended for use in medical applications are single use and disposable [2–4]. In April, 2020, the U.S. Center for Disease Control and Prevention (CDC) recommended use of face coverings by the general public both indoors and outdoors where physical distancing could not be maintained as new evidence emerged that SARS-COV2 is transmitted through air via aerosols generated during breathing, speaking, sneezing, and coughing [5–8]. However, given high demands for certified PPE required by healthcare workers, the CDC recommended that the public use cloth face coverings and disposable non-certified masks so as not to place further pressure on limited certified PPE supplies [3]. The World Health Organization (WHO) followed suit, recommending mask wearing by the public where there is widespread transmission and when physical distancing is difficult [9–11]. Worsening conditions of the pandemic ultimately led to the implementation of mask mandates in several jurisdictions around the world [9, 12–14]. Laboratory studies on facemask filtration efficiency on manikins [15] or people with respiratory illness [6], systematic review of SARS-CoV-2 intervention strategies [16] and population-based modelling studies [8, 17–21] support the use of mask mandates as a means of reducing COVID-19 transmission.

The recommendation of public use of face coverings by the CDC and WHO led to the development of a new cottage industry of cloth mask production and the emergence of home-sewist mask production. Many of these cloth masks were donated to health care institutions and to people in need. However, non-commercial home sewn masks lack the standardization of materials and design leading to uncertainties in their real-world performance relative to certified medical masks. As such these types of face coverings are recommended for use by the public under low-risk, non-professional applications [22] in conjunction with multiple health and safety precautions that include maintaining physical distancing, frequent hand hygiene, and adherence to local health advice and restrictions including indoor building capacity limits, participation in COVID-19 testing programs, contact tracing and self-isolation, and community vaccination programs. Prior to the pandemic there were limited studies exploring performance characteristics of common commercial fabrics that the public could source and use in the construction of home-made cloth masks as alternatives to commercial PPE [23–31]. Notable early studies examined material filtration efficiencies for bacteria and virus-sized particles [23, 24] and quantitative fit testing of constructed cloth masks that focused on the protection of the mask wearer [24–26]. Since the start of the pandemic, more than 40 studies have been published documenting fabric filtration performance and cloth mask filtration efficiency. As such, an entirely new class of mask research literature has developed focusing on masks as barriers against expelled droplets and aerosols, changing the perspective from masks as protection for the wearer to masks as a community source transmission mitigation tool [13, 14, 32–34].

Both the CDC and WHO recommend using cotton as a type of fabric that can be used in home-sewn masks and face coverings, to reduce COVID-19 transmission in community settings [13]. Cotton fabrics have several desirable properties for use as cloth masks. First and foremost, cotton is the most widely available fabric in the world making sourcing and procurement simple for most individuals both in developed and developing nations [35]. Cottons are hypoallergenic [35], they can be ironed (useful for shaping fabric elements during mask construction), and they can be machine washed over numerous washing cycles, making cotton-based cloth masks more sustainable than disposable commercial PPE and some types of synthetic fabrics [4]. Cottons are also generally easy to work with and sew using standard sewing equipment available to most home sewists. Finally, cotton fabric can be obtained in a vast array of colors, textures and prints, lending itself to artistic expression in created cloth masks. Although mask aesthetics do not contribute directly to mask performance, aesthetic attributes of worn garments are likely to inspire greater compliance of mask wearing [36].

While cottons are rarely rated as among the most efficient filtration materials for aerosolized particles, some types of cottons have the potential, especially when used in multi-ply or multi-fabric mask designs, to be considered moderately-effective filtration barriers when paired with good mask design [24–26, 37–41]. Clase et al. [11] and Kwong et al. [42] reviewed fabric filtration studies and after considering operational differences in filtration measurements such as particle type, particle sizes and flow velocity, showed that cottons have a broad and highly-variable range of potential aerosol filtration efficiencies from <5% to as high as 90% depending on material, particle size, face velocity and number of layers used.

Beyond filtration efficiency, materials used in mask construction must be suitable for sewing [24] and be capable of being fashioned into a mask that fits snugly to the face and minimizes leaks along the mask edges [14, 26, 39]. Quantitative fit testing using standard methods such as those that employ a TSI PortaCount Fit Tester in conjunction with a particle generator provide a combination of mask filtration and mask leakage testing when used with constructed masks fitted to volunteers [14, 24, 26, 34]. Such tests typically report mask performance against polydisperse sodium chloride particles in the size range of 0.02–1 μm that are used as surrogates for free viral particles (~0.05–0.15 μm) and the smaller end of the range of viral-laden aerosols emitted by humans during breathing, coughing and sneezing [11, 34, 42]. Although currently-available standardized quantitative fit tests do not detect mask performance for aerosols > 1 μm or droplets which can be up to 1 mm, the smaller particles detected in such tests are considered among the most penetrating of particle sizes [11, 43] and thus quantitative fit tests yield a conservative estimate of mask performance that likely underestimates the full capability of the mask across the entire range of contaminated particle sizes it may be subjected to. Quantitative fit testing of cotton masks has been reported in past and recent studies showing fitted filtration efficiencies in the range of 20 to 98.5% [24–26, 34, 39, 44] for various designs of 100% cotton masks with 2 or more fabric layers. The high variation in both flat-fabric and constructed-mask filtration studies indicates that careful selection of cottons is needed when selecting such fabrics for use in home-sewn masks.

Cottons do possess some disadvantages over synthetic materials when used in cloth masks. Their hydrophilic nature means they absorb moisture and therefore are less suitable as a face fabric barrier against fluids and droplets [4], although as an interior layer, the same hydrophilic property of cottons is viewed as an asset for intercepting droplets generated by the wearer [13, 44]. Hydrophobic synthetic fabrics provide stronger fluid barrier protection, and the high surface tension of fluids at pore perimeters increases the energy required for droplets to penetrate pores, improving their filtration efficiency [42, 45]. In addition, many synthetic fabrics can hold an electrical charge that aids in the retention of very small, nanometer-sized particles that are particularly difficult for cottons and other natural fabrics to remove [45]. As such, a number of researchers recommend masks be made out of a combination of hydrophobic (e.g., polyesters and polypropylene) and natural fabrics such as cotton [13, 22, 23, 42, 44, 45].

Although a growing number of studies have reported on filtration efficiencies for aerosolized particles by different types of cottons [4, 22, 38, 41, 42, 45–48] and cotton mask quantitative fit testing [24–26, 34, 39, 44], many of these studies have tested only a small number of cottons as part of wider exploratory analysis of different material types that include both natural and synthetic fabrics. Furthermore, it can be difficult for home sewists who want to construct their own cloth mask to identify and source high-performance filtration materials given that fabric characteristics described by researchers are often not identified on labels of consumer goods, or when such materials are procured as used goods without any kind of consumer label information attached to them [42, 49].

The objective of the present research was to characterize a large number of commercially-available cotton fabrics according to their potential aerosol filtration performance and breathability for use in the construction of cotton-based cloth masks. The characterized fabrics were categorized into easily recognizable consumer fabric groups so that home sewists, volunteer groups and non-commercial organizations involved in cloth mask making could easily identify, select and source appropriate cotton materials for incorporation into cotton-only or mixed fabric cloth masks intended for personal use or for donation to the general public and vulnerable populations, i.e., those without access to commercial masks or face coverings. Material characterization included fabric pore size measurements, thread characteristics examined by environmental scanning electron microscopy (ESEM), and fabric weight. A subset of the characterized materials were used to construct 2-ply cotton masks of a common design and their breathability and constructed-mask filtration efficiency were tested by differential pressure tests and quantitative fit testing. Statistical models were then developed to predict fabric breathability and 2-ply cotton mask fitted filtration efficiency for each material included in the study.

## Materials and methods

### Fabric source and characterization

Fifty-two cotton fabrics or cotton-based commercial goods were purchased at local stores operating in Windsor-Essex, Ontario, Canada. Purposive sampling was used to include multiple items across nine broad consumer fabric categories identified in Table 1. All items purchased were labelled or marketed at the retail shop as 100% cotton. Commercial sources and branding information on labels for each item is provided in S1 Table. Some of the fabric items represent commercial goods sold for use as-is (e.g., T-shirts, tea towels and bed sheets) while others were purchased as unsewn fabric (1 m of each) with different categorizations of intended sewing applications. For fabrics used as commercial goods or purchased as raw fabric, each item consisted of a single fabric layer.

**Table 1:** Consumer fabric categories, purchase source and intended fabric application of items characterized.

Fabric Category	Number of Different Examples Purchased	Commercial Sources	Intended Use/Application
Bandana	5	convenience store, outlet stores, workwear clothing store, arts and crafts store	headwear, sun protection, consumer good
T-shirt	5	workwear clothing store, department store, arts and hobby store, dollar store	clothing item, consumer good
Fashion Fabric	5	chain operated fabric store	sewing material mainly for constructing clothing
Mass Market Quilting Cotton	7	arts and hobby store, department store, specialized fabric store, chain operated fabric store	sewing material for quilts or clothing, generally lower cost than ‘high quality’ items
Home Decoration Fabric (Subsequently referred to as Home décor)	5	chain operated fabric store, furniture/upholstery shop	sewing material for upholstery, drapes and other cloth based items
Tea Towel	7	big box department store, department store (3 samples), dollar store (2 samples), quilt shop	towel used for drying dishes, consumer good
Bed Sheet	6	big box department store, department store, outlet bedding store	consumer good. For some samples, the pillow case was used after verifying it was the same material as the bed sheet
High Quality Quilting Cotton	7	quilting shop	sewing material for quilt construction and fabric crafts
High Quality Batik Quilting Fabric	5	quilting shop	sewing material for quilt construction and fabric crafts

**Fig 1:**
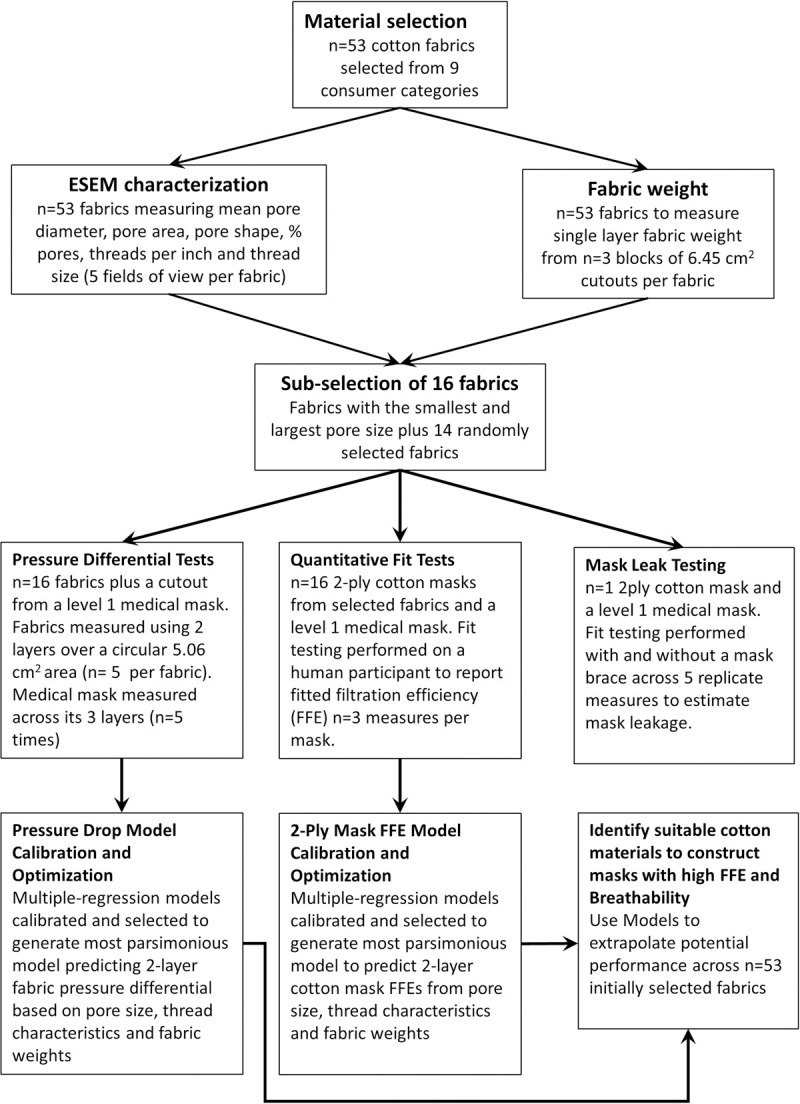
Flow chart of study design used for material and mask characterizations, model calibration and selection of optimum cotton materials for use in constructing 2-ply cotton masks.

Each item was given a unique identification number and photographed and washed using a standard home washing machine using the bedding cycle with laundry detergent and then dried in a home clothing dryer at high heat until fully dried prior to using it for material characterization or mask construction. Item washing was completed using a Samsung home washing machine (Model #WA54M8750Hv/A4) using settings: bedding cycle, hot, medium spin, normal load, extra rinse. The total wash time was 123 minutes. A Hobo Tidbit (OnSet, Cape Cod, MA, USA) temperature probe (UA-002-08) was wrapped in a sock and added to a loaded wash cycle to log temperatures at 1 minute intervals during an equivalent wash cycle. The mean±standard deviation washer material temperature was 32.6±16.8°C, with a peak temperature of 52.7°C reached after 22 minutes and a total of 26 minutes wash time where temperature exceeded 50°C. Drying was completed on an electric powered home dryer (Samsung Model DV422EWHDWR/AC) using the settings: Bedding, Very Dry over a 127 min duration. The mean±standard deviation dryer temperature was 41.6±9.1°C. Temperatures steadily rose during the drying cycle reaching a peak of 55.3°C at the end of the cycle. A flow chart providing an overview of the experimental design and methods applied across the different cotton fabrics is provided in Fig 1.

Three 2.54 x 2.54 cm squares were cut from each item using a new rotary blade cutting tool and placed into a labelled plastic bag for characterization by environmental scanning electron microscope (ESEM). Where applicable, the orientation of the bias was marked on the sides of each square using a permanent marker. The remaining fabric was placed in a separate labelled bag for use to construct masks for selected samples. A set of five disposable face masks with ear loops meeting level 1 standards of ASTM F2100-21 for use by health care workers as personal protective equipment, made in China and distributed by Polar Bear Canada Corp., (henceforward referred to as L1 medical mask) were donated by Hamilton Health Sciences for use as the control medical mask. Four of the L1 medical masks were cut to remove a single 2.54 x 2.54 cm square from each mask. The surface, middle and inner layers from the 3 ply L1 medical masks were separated and characterized be ESEM separately. The fifth mask was used in quantitative fit testing as described below and another used to determine material breathability across the 3-ply material in the face of the mask.

The ESEM (FEI Quanta FEG SEM running in Low Vacuum, 70 Pa chamber pressure) at the Great Lakes Institute for Environmental Research, University of Windsor, was used to perform fabric characterization measurements. One randomly-selected fabric square was taped to the ESEM stage with care taken to not stretch the fabric beyond its normal relaxed state and orientated in a consistent way according to the fabric bias markings. Magnification was fixed at 40x for each sample. Five images were taken for each fabric square by randomly re-positioning the stage to generate 5 different fields of view for each fabric sample. Fields of view were selected so that they did not approach the edges of the fabric square. The ESEM Imaging software (Scandium SIS Version 5 Software) was used to compute fabric metrics described below.

Threads, or yarns, were defined as intact, readily visible distinct bundles of fibers. For most fabrics, threads were oriented in a uniform weave pattern. The total number of completely imaged threads running across the field of view in the horizontal and vertical plane (i.e. warp and weft) were counted and the distance (μm) between the outermost portion of the first and last threads were measured using the imaging software. This was converted into the number of threads per inch (TPI) in each direction as the sum of horizontal and vertical threads per inch. Some fabric images showed discontinuities in the thread weave and therefore the choice of which horizontal or vertical line of threads counted in a given field of view was at the analyst’s discretion, based on what appeared to be the most representative of the fabric sample. For thread size, two randomly-selected threads from each field of view were measured as the distance (μm) across the width of the thread or distinct bundle of fibers. Thread sizes were reported as the mean ± standard deviation of 10 threads measured in each of the horizontal and vertical directions. Pores were defined as gaps or holes present between threads and visually represented as black regions on the ESEM image. A colorization filter was used to aid in pore identification. The analyst used the imaging software to generate a polygon outline of 10 selected pores for each field of view to generate 50 pores characterized for each fabric. Effort was made to select pores with varying size. Pores were selected to avoid those bisected by stray fibers. For each polygon, the imaging software computed the mean pore diameter (μm), pore area (μm^2^) and shape factor (value ranging from 0 to 1, with 0 representing a perfectly straight line and 1 representing a circle). The imaging software also reported the total area and % of field of view occupied by black regions on the ESEM image corresponding to the % area of pores present in the fabric. The percent pores is reported as the mean ± standard deviation of pore percentages across 5 fields of view. Fabric weight was measured by weighing each of the triplicate 2.54 x 2.54 cm fabric squares using a 0.1 mg analytical balance and expressing the mean ± standard deviation weights in units of g/m^2^. Given that all fabrics, except for the medical mask control layers, were cotton, fabric weight is expected to be strongly related to fabric thickness. Given the distinct differences in the construction of the medical mask, only pore size was reported for the individual layers of this fabric type.

A total of 17/53 fabrics from across the fabric samples were selected to create cloth masks for quantitative fit testing. The chosen design was the Essex Pleated mask, a two-layer, 3 pleated design created and refined by members of the Windsor-Essex Sewing Force (WESF), a community group focused on making homemade cloth masks for donation to vulnerable people. The mask construction pattern and instructions for the Essex Pleated Mask design is provided in S1 File. All of the cloth masks used fabric ties as a head attachment. For the chosen design, the ties thread freely through vertical channels at the side of the mask which enables self-adjustment of the mask fit when tying the fasteners behind the head. The fabric samples used for mask construction were selected following ESEM characterization and preliminary analysis of ESEM results. Two fabrics were selected to represent the fabric with the smallest and largest mean pore sizes. The remaining 15 fabrics were selected by randomization using Microsoft Excel random number generator (RandBetween function with removal of duplicate numbers). All masks were constructed by the same sewist who had extensive experience sewing hundreds of cloth masks of the chosen design as a volunteer with WESF. One of the selected fabrics (tea towel, i.d. #WP029) could not be made into a mask because the material was too thick to sew. Sewist comments on the ease of construction of masks are provided in S1 Table. The remaining completed 16 masks were inspected by an independent sewist who verified that each mask conformed to the specified dimensions and retained critical mask features such as retention of pleats and overall quality of construction.

### Differential pressure testing

The same 16 fabrics selected and used to construct 2-ply cloth masks described above were characterized by differential pressure testing for breathability. Differential pressures were measured according to the British Standards Institution (BSI) guidelines identified by BS-EN 14683–2019 Annex C, which is the approved test procedure for ASTM-F2100-21. For each selected material, 2 layers of the same fabric, ‘wrong’ sides together, ‘right’ sides out, with warp aligned with warp (i.e., in the same orientation used in a constructed cloth mask), were clamped into the testing apparatus exposing a circular portion of the fabric layers with an area of 5.07 cm^2^ across the orifice separating the test chambers. A vacuum was applied until atmospheric air was drawn through the sample material to a rate of 8 L/min as indicated by an in-line rotameter (Terra Universal, Fullerton, CA). The differential pressure is read from an inclined differential manometer (Dwyer, Michigan City, IN), converted into units of mm H_2_O and then normalized by the fabric’s surface area in cm^2^. Separate pressure differential measurements (n = 5) were taken for each set of fabrics. Based on breathability guidelines applied to level 1 medical masks, a pressure differential of 5.0 mm H_2_O/cm^2^ is recommended as the upper limit (ASTM-F2100-21). Five additional pressure differential readings were measured across the 3 fabric layers of the medical mask used as a control.

### Quantitative fit testing

Quantitative fit testing measures a combination of the aerosol filtration efficiency of the material incorporated in the mask and the tightness of the seal of the mask to the participant’s face. As such, quantitative fit testing gives a ‘real world’ performance measure of the mask when tested on human participants whereas material filtration tests only provide the filtration efficiency of the material in question and may not predict the actual performance of a constructed mask.

A TSI 8038 PortaCount Plus Respirator Fit tester set up at the Department of Engineering, McMaster University, Hamilton, Ontario was used to perform quantitative fit testing on cloth fabric masks and a control L1 Medical Mask. Both participants gave informed consent and the study was approved by the Hamilton Integrated Research Ethics Board. A TSI 8026 particle generator was used to produce NaCl aerosol particles in the enclosed testing room to generate a size range of 0.02–0.6 μm polydisperse particles with a specified median particle diameter of 0.05 μm. These particles supplemented naturally-occuring particles in the room. During fit testing, the PortaCount cycles between an ambient air intake located outside, in front of the mask, and another intake that samples air inside the mask connected through the mask by a sampling port rivetted through the mask material. Testing was performed according to the Canadian Standard Association (CSA Z94.4–2002 protocol) which averages ambient air/inner mask air particle concentrations taken across a set of 7 activities, each performed for 34 seconds: normal breathing, deep breathing, nodding head from side to side, nodding head up and down, talking out loud, bending forward and return to neutral position and normal breathing again. The testing protocol is similar to Occupational Safety and Health Administration (OSHA) 1910.134 but with a shorter testing duration for each activity. We tested in ‘all particles’ mode, which disengages the particle classifier and detects and reports all particles (salt and naturally-occurring) in the range 0.02–1 μm. During mask testing, the mean ambient particle concentration was 5167 particles/cm^3^ and ranged from 4380–6428 particles/cm^3^ across individual tests. Ambient particle concentration at the beginning of the test was not significantly related to fitted filtration efficiency (FFE) of the tested masks (ANOVA; p>0.9). The fit factor (FF) is defined as the ratio of particle concentration in the ambient air to particle concentration in the interior of the mask, for each activity, and the overall FF is expressed as the geometric mean FF across 7 activities was computed automatically by the PortaCount software and manually recorded after each test. The overall fit factor was converted to a fitted filtration efficiency (FFE) according to Eq 1.


$$FFE(\%)=\left(1-\frac{1}{FF}\right)∙100$$ (1)

Fit testing was completed on the 16 constructed fabric masks and on the L1 medical mask. Fit testing of the 16 constructed masks plus control were conducted on the same person, tester #1 (male 89 kg, 178 cm, no facial hair). The order of mask testing was randomized by Microsoft Excel random generator. Between each test, the selected mask was donned and tied in place by the mask wearer with care taken to ensure a consistent fitting of each mask. Each mask was tested three times in random sequence and donned and doffed separately for each test. The L1 mask worn with integral ear loops was tested 4 times with replicates intermixed between cloth mask tests. Masks were tested with the pleats directed down.

Recognizing that quantitative fit testing simultaneously measures both material filtration and fit (i.e., loss of efficiency due to leaks), an additional set of fit testing measurements was conducted on one of the fabric masks (WP045) and the L1 medical mask to test mask performance with and without a mask fitter. The mask fitter, or mask brace, consists of a molded elasticized strap that holds the mask tight to the mask wearer’s face. Its function is to reduce leaks through the top, sides and bottom of the mask. The brace used was a commercially available product called “Fix The Mask” which has additional cushions that help secure the mask around the nose. Mask fitters help achieve fit testing results that approach the theoretical maximum filtration efficiency of the mask [14, 26, 39]. The mask fitter test was conducted in order to evaluate the impact of poor fit on mask performance recognizing that the medical mask and the constructed cloth masks differed in a number of important ways, related to fit: the medical mask had a bendable nose strip and elasticized ear loops while the cloth masks did not contain nose wires and were fixed to the tester’s head by use of fabric ties around the back of the head. For this subset of tests, the L1 medical mask and WP045 Fabric Mask were tested 5 times by a tester #2 (female, 80 kg, 176 cm). Each mask was tested in sequence using the same fit testing methodology described previously. Between each test, the mask was doffed and redonned again and tested without brace first then tested with the brace until all five replicates of each treatment and mask type were completed. Mask leakage was estimated for both the L1 medical mask and WP045 as the difference in mean FFE with and without the mask fitter based on tester #2 results.

### Data analysis

Summary data by fabric category are reported as fabric means and standard errors along with the coefficient of variation (CV, %; calculated as the standard deviation divided by the mean multiplied by 100%). Across the fabric measurements, principal components analysis (PCA) was used to evaluate intercorrelations between the individual fabric measurement metrics. A correlation matrix was used for the PCA analysis and PCA results were interpreted using a biplot that presents fabric measurement loadings as vectors and mean fabric scores for each consumer category across the significant PCA axes. Analysis of variance (ANOVA) with Tukey’s pairwise comparisons was used to test for differences in mask fitted filtration efficiencies between individual masks and the L1 medical mask after verifying normality assumptions using Shapiro Wilks test. For material breathability measurements, ANOVA and Tukey’s pairwise comparisons were used to test for differences in the log_10_ transformed differential pressure between each material tested and those of the Level 1 medical mask. Log transformation of the data was required to satisfy assumptions of normality (p>0.1; Shapiro-Wilk test). Probabilities less than 0.05 were deemed the criteria for statistical significance.

Multiple regression models were used with forward and backward stepwise options to generate a predictive model of fitted filtration efficiency (FFE, dependent variable) from the various fabric measurements used as independent variables. The initial predictor variables included pore diameter, pore area, percent pores in fabric, TPI, horizontal thread size, vertical thread size, and fabric weight. Model optimization removed the least predictive variables based on the lack of statistical significance of the coefficient when included in the model until only significant variables remained. After completing the backwards stepwise regression, a new model was tested that consisted of the significant predictor variables plus variable interactions. Model parsimony was checked by minimizing the number of variables included in the model and comparing Akaike’s Information Criteria (AICs) computed with multiple regression model fits. By rule of thumb, if the AIC of the model with one additional variable is lower by more than 3 units compared with the simpler model, it is considered a better model. A similar exploratory process was performed by substituting fabric measurements as predictors for PCA scores across the significant PCA axes. The strongest performing model was subsequently used to predict fitted filtration efficiencies for the remaining fabric materials that had not been used to construct and test masks. The same model calibration and optimization procedure was applied to material pressure differential results and used to estimate material breathability across the remaining untested fabric materials. One-way t-tests were used to determine the probability of exceeding the ASTM-F2100-21 Level 1 medical mask breathability guideline value. For these tests, model-extrapolated breathability values for individual fabrics were grouped into fabric consumer categories and used to test the hypothesis that the mean differential pressure of materials within the consumer category exceeds a value of 5.0 mm H_2_O/cm^2^. All statistical analyses were completed using SYSTAT Version 13 statistical software.

## Results

### Fabric characterization

**Fig 2:**
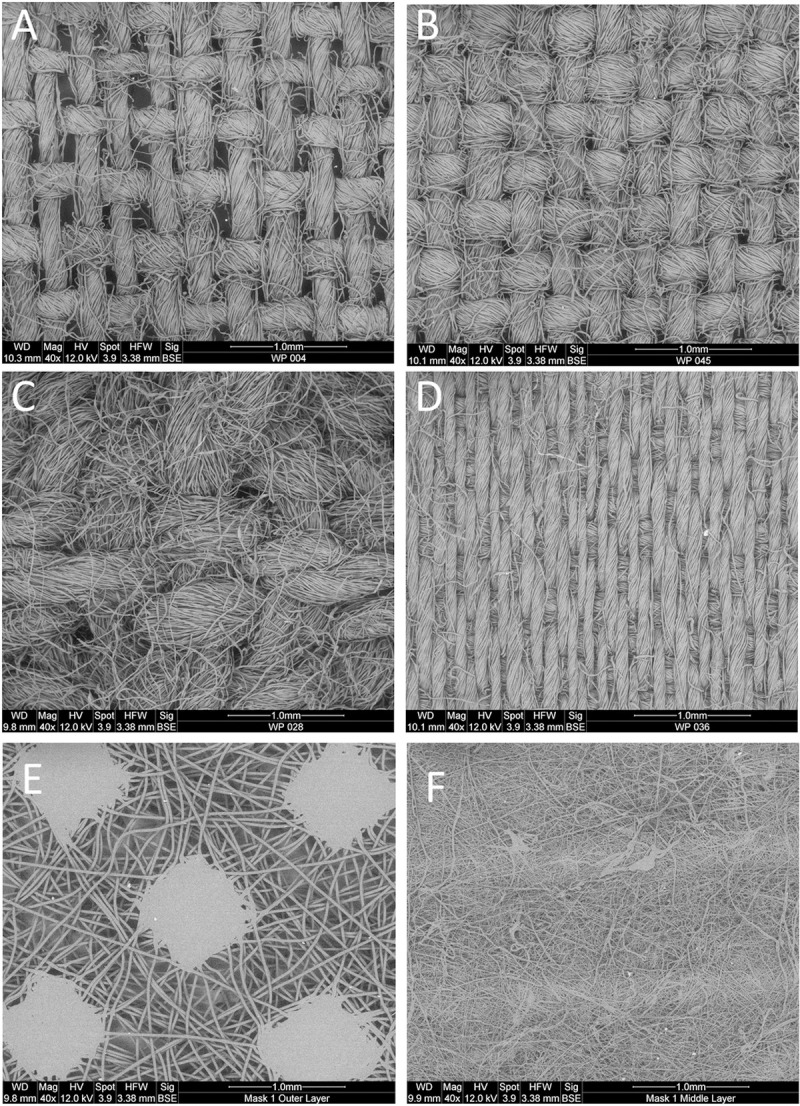
Electron microscopy images of selected fabrics. A) WP004 Bandana with plain weave, B) WP045 High Quality Quilting Fabric with plain weave, C) WP028 tea towel with complex weave; D) WP036 Bed Sheet with complex weave, E) Outer Layer L1 medical mask nonwoven, F) Middle Layer of L1 medical mask nonwoven. Microscope images for all 52 fabrics are provided in S2 File.

**Table 2** provides summary data on selected fabric characteristics averaged across the nine fabric consumer categories. Detailed information about each of the fabric measurements for all fabrics tested are also provided in S1 Table. Values for TPI ranged from 72.6 threads/inch (tea towel; WP031) to 325.7 threads/inch (bed sheet; WP040) and were generally lowest for T-shirts and highest for bed sheets. Mean fabric pore diameters ranged from 38.9 μm (bedsheet; WP036) to 199.4 μm (bandana, WP004). Mean pore diameters were generally less than 100 μm for several fabric types including high-quality batik cottons, bed sheets and high-quality quilting cotton but were larger for bandana and T-shirts. For the L1 medical mask, mean pore diameter approached 100 μm for the outer and inner layer fabrics, but was much smaller (20.1±0.64 μm) for the middle layer. Pore areas by fabric category had a strong log linear relationship with mean fabric pore diameter resulting in similar fabric rankings. The total area of fabric occupied by pores ranged from <1% (mass market quilting cotton; WP020) to as high as 18.5% (T-shirt; WP-008). Bandana, T-shirt and tea towel fabric categories exhibited the largest % pores averaging 8 to 12% while bed sheets and high-quality batiks were among the lowest (Table 2). The L1 medical mask had mean (±standard error) % pores of 10.6±1.1%, 10.4±1.6% and 11.3±1.6% for the outer, middle and inner layers, respectively. Fabric weights ranged from 64.5–344.5 g/m^2^ across fabric types. Fabric weights were greatest for tea towels, home décor and T-shirt materials and lowest for bandana. Fig 2 presents selected images of 4 fabrics with large (WP004, bandana), moderate (WP045, high-quality quilting cotton), the smallest pore size (WP036, bed sheet) and with the highest fabric weight (WP028, tea towel) contrasted with the non-woven outer and middle layers of the L1 medical mask. ESEM images and non-magnified fabric sample photos are further provided for each fabric type in S2 File. Table 2 also presents coefficients of variation (CV) for fabric measurements to provide information about variation of individual fabrics within a given consumer category. Despite performing well across a number of metrics, bed sheets as a consumer category had the highest CVs for thread density, pore diameter, pore area and % occupied area. High quality quilting cottons had the lowest CV across fabrics tested for 4 of the 5 measurement categories.

**Table 2:** Selected mean ± standard error (%CV) of fabric characteristics across fabric consumer categories.

Category	Threads Per Square Inch	Pore Diameter (μm)	Pore Area (μm^2^)	% Area Occupied by Pores	Fabric Weight (g/m^2^)
Bandana (n = 5)	150.1±7.3 (10.8%)	151.1±13.6 (20.2%)	10522±1813 (38.5%)	7.98±0.30 (8.5%)	93.0±7.6 (21.5%)
T-Shirt (n = 5)	81.9±2.1 (5.7%)	139.3±14.9 (24.0%)	7349±1441 (43.8%)	12.54±1.73 (30.8%)	185.2±10.5 (14.9%)
Fashion Fabric	165.8±15 (20.4%)	115.8±10.5 (20.3%)	5765±1108 (43.0%)	4.94±0.85 (38.6%)	112.5±10.9 (25.6%)
Mass Market Quilting Cotton	150.7±11.4 (20.1%)	111.8±4.7 (11.0%)	5593±648 (30.7%)	4.35±0.67 (40.6%)	131.7±5.9 (14.4%)
Home Decor	110.3±13.9 (28.2%)	110.8±8.4 (17.0%)	4532±451 (22.3%)	4.95±0.64 (29.0%)	208.4±18.0 (22.7%)
Tea Towel	95.1±6.3 (17.5%)	145.4±10.0 (18.2%)	8837±1121 (33.6%)	12.34±1.74 (37.2%)	239.5±18.1 (24.3%)
Bed Sheets	249.1±26.9 (26.4%)	78.1±10.8 (33.9%)	2601±624 (58.8%)	2.26±0.72 (77.7%)	123.9±4.6 (11.0%)
High Quality Quilting Fabric	134.6±1.8 (3.6%)	91.5±2.8 (8.1%)	3796±221 (15.4%)	3.59±0.59 (43.1%)	157.9±1.3 (2.7%)
High Quality Batik Fabric	217.2±0.8 (0.8%)	67.0±5.7 (18.9%)	1752±380 (48.6%)	1.93±0.22 (25.7%)	121.9±6.2 (13.4%)
L1 Surface	NA	100.1 ± 3.9	4649±329	10.59±1.12	NA
L1 Middle	NA	20.1±0.6	190±17	10.41±1.58	NA
L1 Inner	NA	98.4±3.1	4390±207	11.28±1.59	NA

**Fig 3:**
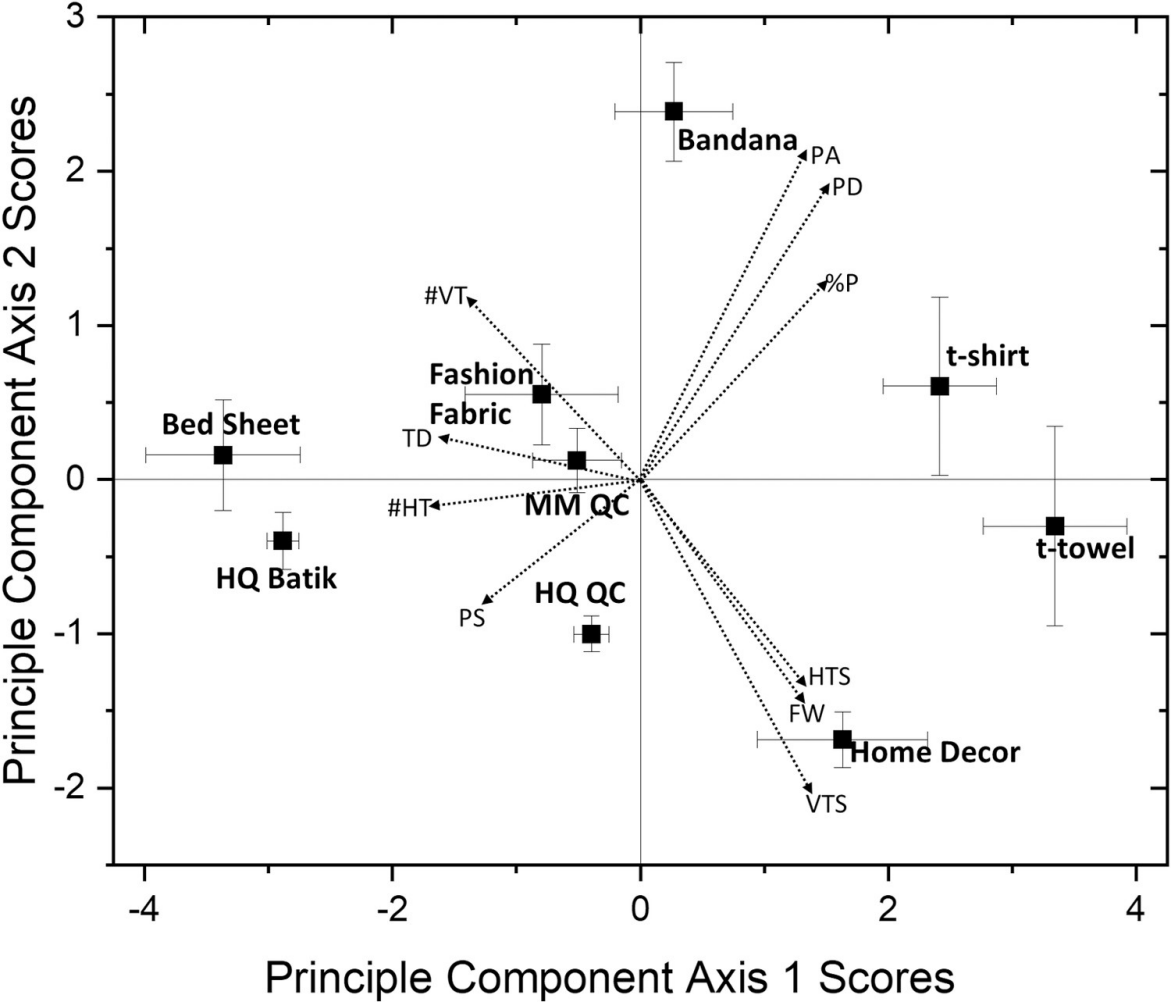
Biplot of principle component scores and fabric measurement vectors. PA = pore area; PD = pore diameter, %P = % pores in fabric, HTS = horizontal thread size, FW = fabric weight, VTS = vertical thread size, PS = pore shape, #HT = number horizontal threads, #VT = number of vertical threads, TD = thread density.

Principle components analysis (PCA) on the cotton fabric measurements generated two component axes with eigenvalues greater than 1 and which cumulatively explained 76.8% of the variation in the data. Fig 3 presents a biplot of PCA scores across the first two PCA axis with samples grouped according to their fabric type while vectors present fabric measurement loadings. Fabrics positioned towards the left upper and lower quadrant exhibited generally smaller pore sizes and higher TPI. These included bed sheet and high quality batik at the extreme range followed by clustering of high quality quilting cotton, mass market quilting cotton and fashion fabric. Distinction between the upper and lower left quadrant of Fig 3 was mainly according to pore shape and number of vertical threads. Fabrics falling in the upper right quadrant included bandana and T-shirt were characterized by larger pore sizes and % pores. The tea towels and home decor fabrics were positioned towards the lower right quadrant and generally characterized by a combination of large pore size, large thread size, but also higher fabric weights.

### Quantitative fit testing of fabric and medical masks

Sixteen Essex Pleated fabric masks were constructed from selected fabric samples and used to measure fitted filtration efficiency (FFE) by quantitative fit testing. Each mask, along with the L1 medical mask was tested in triplicate on the same mask user. There were highly significant differences (p<0.001; ANOVA) in FFEs between the tested masks. The control medical mask generated a mean ± standard error FFE of 55.3±2.1%. The best performing fabric mask was WP036, made from a bed sheet, that generated a mean ± standard error FFE of 65.6±4.6% followed closely by WP028 (tea towel; 65.0±1.4%) and WP047 (batik; 64.3±0.7%). The three bandana masks yielded the lowest FFEs ranging from 39.8 to 48.1% and as a group, bandanas generated significantly lower (p<0.01; Tukey’s pairwise comparison) FFEs compared to the L1 medical mask. All other tested fabric masks yielded FFEs that did not significantly (p>0.05; Tukey’s pairwise comparison) differ from the medical mask.

**Fig 4:**
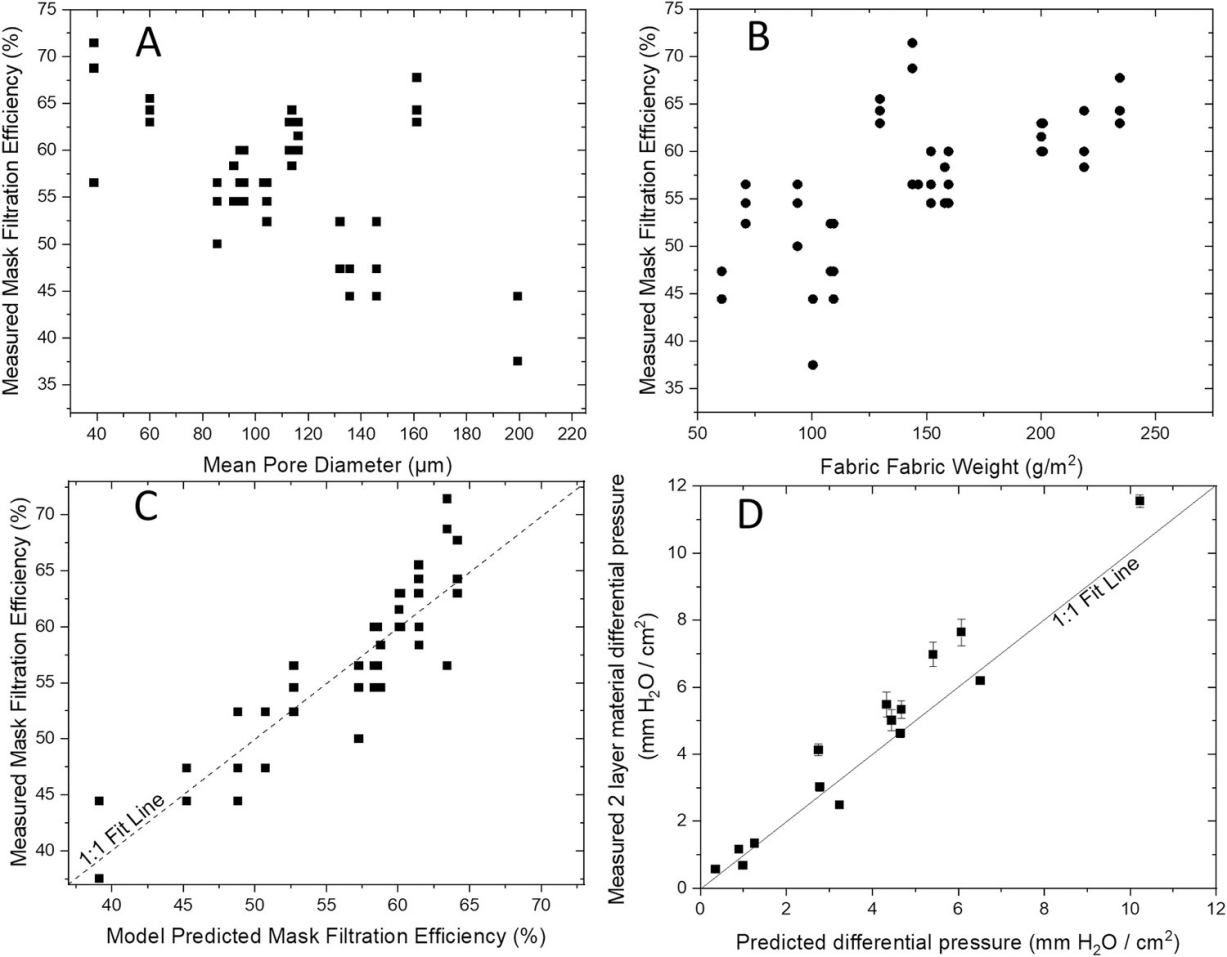
Correlation between mask filtration efficiency and mean fabric pore diameter (**Graphic A**) and fabric weight **(Graphic B**) and goodness of fit tests comparing observed against predicted mask performance from Eq 2 (**Graphic C**) and for material differential pressure from Eq 3 (**Graphic D**).

Several models to predict FFE from fabric characteristics were explored using multiple regressions by examining different combinations of highly predictive variables along with their interactions. Measurements of pore size, % pores in fabric and fabric area density were among the best predictors. Fig 4 demonstrates the negative relationship between fabric pore diameter and FFE (top graphic) and positive relationship between fabric weight and filtration efficiency (middle graphic). The most parsimonious model, i.e., the model with the smallest number of predictors and highest coefficient of determination (R^2^), was one that included pore diameter, fabric weight and the pore diameter x fabric weight interaction term. The optimized model (Eq 2) had a coefficient of determination (R^2^) of 81% and the goodness of fit test is provided in Fig 3 (bottom graphic).


FiltrationEfficiency=−0.31±0.05∙PoreDiameter−0.093±0.05∙FabricWeight+0.0015±0.004·(PoreDiameter·FabricWeight)+80.65±6.56 (2)

**Fig 5:**
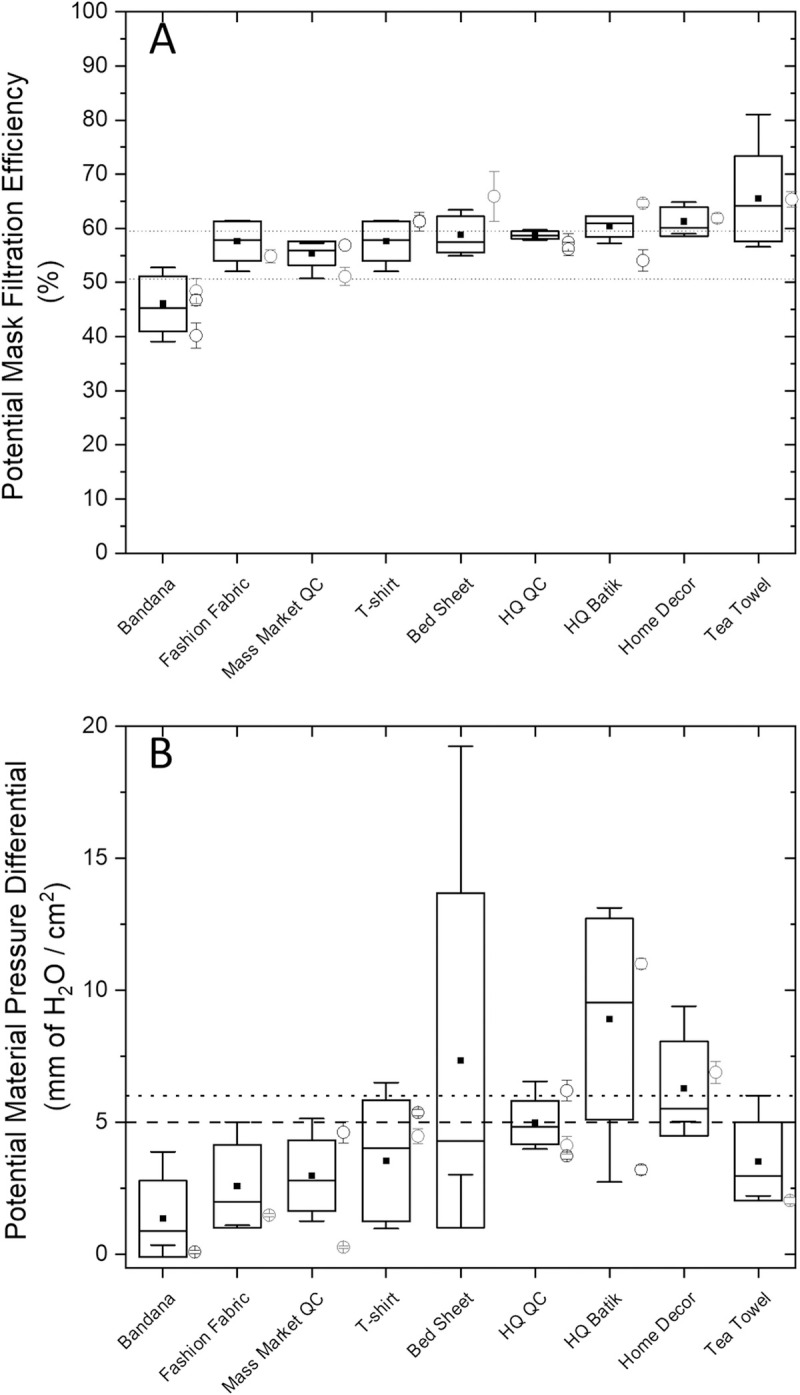
Model estimated mask performance across consumer fabric categories (**Graphic A**). Box and whisker plots present the median (box center horizonal line), mean (▪), standard deviation (upper and lower box edges) and range (whiskers) of model predicted mask filtration performance across each fabric category. Symbols (○) present mean ± standard error of measured mask fitted filtration efficiency determined for each tested fabric masks from each category. Horizontal dashed lines present the range of measured mask filtration efficiencies for the L1 medical mask**. Graphic B**. Model estimated breathability performance across consumer categories. Graphic legend descriptors similar to those described for graphic A. Dashed horizontal line is the Level 1 Medical Mask ASTM guideline value, dotted line is the Level 3 Medical Mask ASTM guideline value.

Eq 2 was subsequently used to predict the potential mask performance for all 52 fabrics. Fig 5 presents distributions of the expected mask FFEs for different consumer categories along with measured mask performances and the FFE range for the L1 medical mask. All consumer fabric categories, except for bandana, are likely to generate masks that have performances that fall within those achieved by the Level 1 medical mask. The highest predicted performing fabric category was teas towels, mostly due to the very high fabric weight of this fabric compared to others. Home décor, high quality batik, high quality quilting cotton, bed sheets and T-shirt all performed at the high end of the medical mask range, and somewhat lower predicted FFEs (but still acceptable performances) were predicted for fashion fabric and mass market quilting cottons. High quality quilting cotton was notable as having both a relatively high performance but also a very small degree of variation in potential performance across the different samples of fabrics analyzed. Tea towels were among the most variable fabric types but are predicted to perform uniformly well across the 7 characterized tea towel samples. One tea towel, WP029 was predicted to have a theoretical FFE of 81% owing to it having the highest fabric weight (344.5 g/m^2^) but the high fabric thickness of this material prevented it from being constructed into a mask.

### Differential pressure tests for selected 2-ply cotton materials

The same sixteen fabrics used in quantitative fit testing were used to test for material breathability across two layers of the same fabric type. Individual materials had differential pressure measurements that ranged from 0.55 to >12 mmH_2_O/cm^2^. One material, WP036 (bedsheet) which had the lowest pore size of tested materials had a differential pressure exceeding the instrument detection limit of 12 mmH_2_O/cm^2^ across all five independent measurements. Excluding WP036, there were highly significant differences (p<0.001; ANOVA) in material differential pressures between the tested fabrics. The control medical mask (3 ply layer) generated a mean ± standard error differential pressure of 4.84±0.08 mmH_2_O/cm^2^. In addition to the low breathability of WP036, six additional materials (WP006 and WP009, both T-shirts; WP022, a mass market quilting cotton, WP027 home décor, WP042, high quality quilting cotton and WP047, high quality batik) had 2-layer differential pressures that were significantly greater (p<0.05; tukey’s pairwise comparisons) than the 3-layer level 1 medical mask. Four of the above materials had mean differential pressures greater than 6 mmH_2_O/cm^2^ reflecting the upper limit of a level 3 medical mask (ASTM-F2100-20).

Statistical models were evaluated to predict material differential pressures from fabric characteristics similar to those described for FFE. Non-detectable measurements for WP036 were excluded from model calibration. The most parsimonious model, i.e., the model with the smallest number of predictors, highest coefficient of determination (R^2^) and met the AIC criteria, was a model that included pore diameter, pore shape and fabric weight without any interaction terms as described in Eq 3.


$$Log_{10}\;differential\;pressure=‐0.011\pm 0.001\cdot Pore\;Diameter-2.581\pm 1.120∙pore\;shape+0.003\pm 0.001\cdot Fabric\;Weight+2.675\pm 0.792;\;R^{2}=0.92$$ Eq 3

Notably, when Eq 3 was applied to predict breathability of WP036, excluded from the calibration, it generated a very high estimate for this material at 19.24 mmH_2_O/cm^2^. A slightly underestimated value was predicted for WP047 (predicted value = 10.2 compared to measured value of 11.55 mmH_2_O/cm^2^). The linearized goodness of fit test between measured and predicted material differential pressures is provided in Fig 4. Eq 3 was subsequently used to predict material differential pressures for all characterized fabrics as identified in S1 Table. When fabrics were grouped into consumer fabric categories, only high quality batik fabrics had a mean fabric category pressure differential expected to significantly (p<0.05; one sample t-test) exceed the Level 1 medical mask criteria of 5 mmH_2_O/cm^2^. Bed sheets were predicted to achieve the highest average differential pressures, but were also among the most variable fabric types for breathability across samples tested. Thus, even though 2/6 characterized bed sheets had predicted differential pressures as high as 9 and 19 mmH_2_O/cm^2^, four other samples had breathability lower than guideline limits.

### Cloth and medical mask performance with a mask fitter

**Fig 6:**
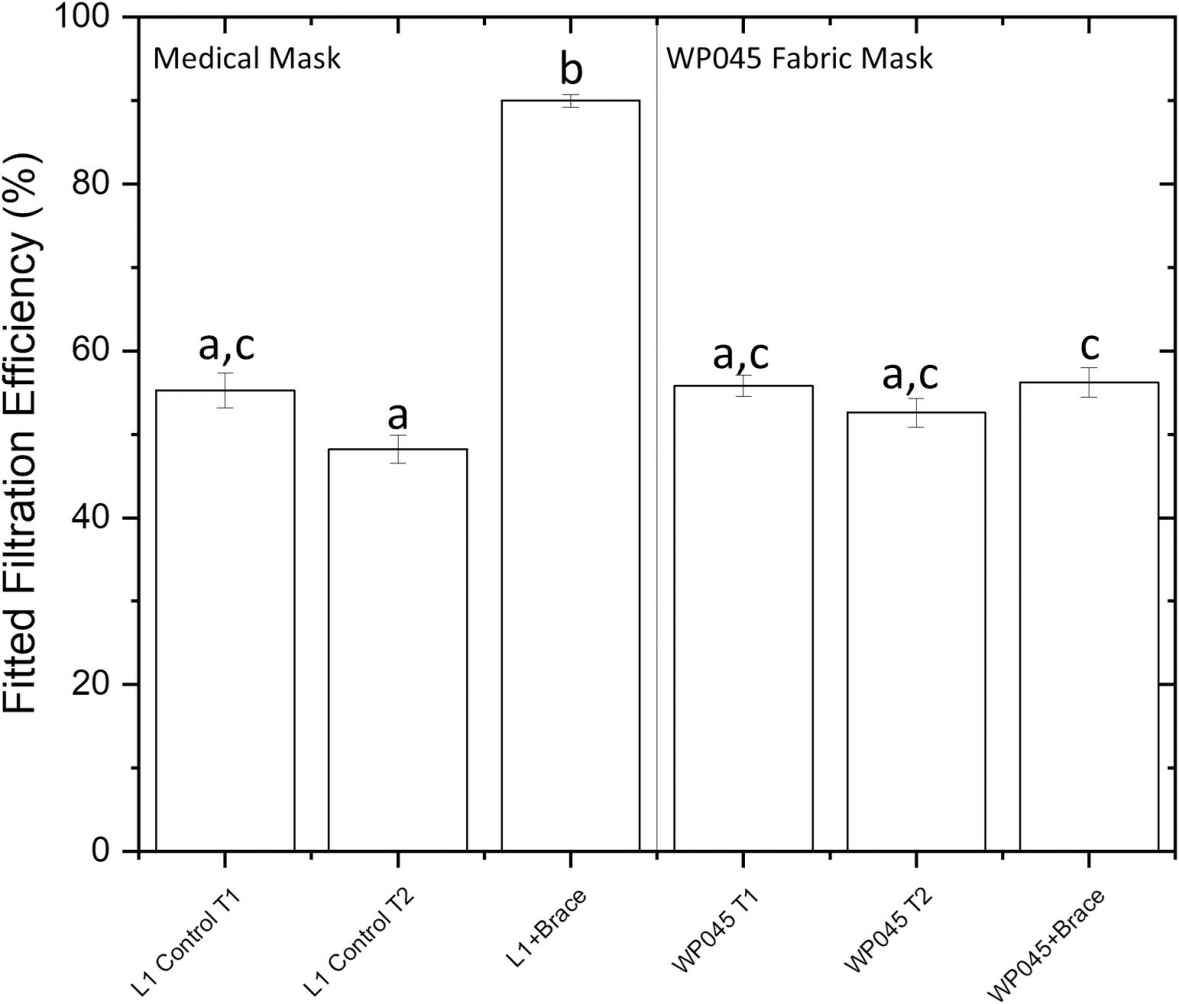
Fitted filtration efficiencies with and without a mask fitter. Column descriptors T1 and T2 refer to Tester #1 and Tester #2. L1 control refers to the L1 medical mask donned with ear loops, L1 + Fitter is the medical mask worn with a mask fitter, WP045 is a high quality quilting cotton mask donned with fabric ties and WP045 + Fitter is the same mask worn with a mask fitter. Bars with different letters are significantly different from one another (p<0.05; Tukey’s pairwise comparisons test).

One selected fabric mask (WP045, high quality quilting cotton) and the L1 medical mask were further used to measure mask filtration efficiency with and without a mask fitter to examine for differences in mask performance that could be attributed to poor fit as opposed to differences in material filtration efficiency. There were highly significant differences (p<0.001; ANOVA) in mask test results across the mask fitter/non fitter contrasts (Fig 6). When the control medical mask was tested with a mask brace it demonstrated a very large and highly significant (p<0.001; Tukeys test) increase in performance with a mean ± standard error FFE of 90.0±0.8% compared to its performance without the mask brace, 48.2±1.7% indicating leakage across gaps from the medical mask as high as 41.8%. In contrast, the Essex Pleated WP045 mask with mask fitter in place did not show a significant (p>0.3; Tukeys test) improvement relative to tests conducted without the fitter with a mean leakage estimate of 3.7%. The fitter/no fitter mask tests were conducted by a different mask wearer (tester #2); both mask wearers had completed replicate FFE measurements on the L1 and WP045 mask without the mask fitter (Fig 5 also includes non-brace FFE for Tester 1 for the L1 Control and WP045 mask). When the mask fit testing results were compared between the two mask wearers (excluding the fitter results), there was a significant difference (p<0.05; ANOVA) in mask performance between testers, with tester 2 generating FFEs that were 3% to 7% lower than those of tester 1. The differences between mask wearers were lost when all contrasts (masks with and without braces and both mask wearers) were compared by pairwise comparisons (Fig 6) reinforcing that the masker wearer effect was relatively small.

## Discussion

### Fabric characterization

The present study characterized 52 cotton fabrics by ESEM generating measurements of mean pore diameter, % pores, TPI, thread size and fabric weights that in general fell within ranges reported for cottons and synthetic fabrics from the PPE fabric-testing literature [4, 12, 13, 37, 41, 45, 46]. Several studies have linked fabric characteristics to aerosol filtration capacity and fabric potentials for use in protective face coverings. Bhattacharjee et al. [13] recommended fabrics used for mask construction have an average thread count greater than 200 TPI which was common among bed sheets and batiks but lower for other consumer fabric categories tested. Konda et al. [45] reported that 600 TPI cotton generated among the highest material filtration efficiencies for cottons tested. However, Crilley et al. [22] reported thread count may not correlate strongly with filtration because higher thread count fabrics can consist of thinner fibers that lead to greater porosity. Indeed, within our study there was no significant relationship (slope not significantly different from zero; p>0.9; ANOVA) between TPI and mask performance. It is also noteworthy that the reported thread counts on consumer labels for bedsheets characterized in the present study bore no relationship with ESEM measured TPIs and therefore caution is warranted when relying on consumer fabric labels and item marketing terms as a means of selecting fabrics used in cloth masks (S1 Table). Bhattacharjee et al. [13] also recommended a porosity of less than 2% which was commonly observed for bedsheets and batiks and some high quality quilting fabrics, but similar to TPI, % pores as a single variable was not a strong predictor of mask performance (slope not significantly different from zero; p>0.5; ANOVA). Pore diameter, a key predictor of mask FFE, ranged from 38 to 199 μm and was similar to the range (77–461 μm) of pore sizes reported by Neupane et al. [31, 49] for face fabrics taken from various commercial cloth masks. Several studies have highlighted the importance of pore size [4, 31, 42, 50] to filtration performance, although pore size itself does not necessarily place a lower limit on the size of particles filtered. This results from multiple particle retention mechanisms; in addition, many masks are made of multiple fabric layers that misalign pores between layers [4, 11, 31, 42]. Model predicted FFEs were most strongly influenced by pore diameter for bed sheets, batiks and high-quality quilting cottons where pore sizes were generally less than 100 μm and fabric weights were low or moderate. For the L1 medical mask, there were distinct differences between the mask layers with the surface and inner mask layers exhibiting mean pore diameter approaching 100 μm, while the middle layer had pore diameters that averaged 19.9 μm in size, considerably smaller than most of the cotton fabrics characterized except one bedsheet sample where the pore size was 38.9 μm.

High fabric weight has been frequently attributed to better material filtration potentials [11, 13, 22, 41, 42, 50]. Fabric weight is related to several fabric properties including thickness, rigidity, drape, air permeability and thermal properties [51] and thus unsurprisingly related to mask performance. The significant interaction between fabric weight and pore size of Eq 2 indicates an interplay between these two fabric characteristics. Increasing fabric weight positively correlates with higher mask FFE’s (Fig 3B), yet the coefficient for fabric weight in Eq 2 is negative and only approached significance (p = 0.055; t-test) whereas a much higher model effect size is generated by the positive and highly significant (p<0.001; t-test) interaction term. This can be interpreted as higher fabric weights compensating for larger pore sizes for some fabrics, possibly a result of discontinuities in surface pore size through the depth of thicker fabrics. According to Eq 2, the relative effect size of the interaction term on predicted FFE’s was most pronounced for tea towel followed by T-shirt and home décor fabrics. These fabric consumer categories were also different in their construction. The tea towel tested was constructed with a waffle weave, T-shirts were all jersey knits while the home décor and some bedsheets were woven fabrics with complex weaves rather than a plain weave (most fabrics tested had a plain weave) (See S1 Table). Although fiber size was not measured, some notable patterns were apparent across ESEM images. T-shirts and tea towels had loose networks of stray fibers that did not appear as tightly incorporated into individual yarns compared to many of the woven fabrics. These networks of loose fibers crossed pores more regularly compared to the plain weaves. Other researchers have commented on the importance of fabric weave, fiber diameter and face-finishing characteristics to fabric filtration potentials [22, 42, 48].

### Quantitative fit testing of cloth and medical masks

Despite the high research interest stimulated by the onset of the global pandemic for fabric mask research, there remain only a moderate number of studies that have performed fabric mask testing on human volunteers using a TSI PortaCount system [24–27, 34, 39, 52, 53]. Comparing fit testing results of cloth masks is complicated by the fact that different masks are constructed with different fabrics, designs, number of fabric layers and utilize different methods for mask attachment. Literature fit testing of 100% cotton masks without filters are described below for comparison with the present research. Davies et al. [24] fashioned a 2-ply mask of cotton T-shirt and observed an FFE of 50% that was slightly lower but within the lower range of predicted T-shirt FFE’s from the present work. Dato et al. [25] described a very high performing mask constructed of 9 layers of pre-shrunk heavyweight cotton T-shirt that yielded FFEs of 92.3–98.5% across three volunteers. van der Sande et al. [26] reported a mean FFE of 60% for adults and 52% for children wearing a tea towel mask that fell within the range of predicted FFE’s for tea towels in the present work. O’Kelly et al. [53] reported quantitative fit testing on 4 fabric masks producing a mean FFE of 52.4%. Bandana material as a face covering varied widely with some studies fashioning bandana into masks and others simply tying the folded bandana across the head and leaving the fabric loose below the chin. Reported FFE’s of 100% cotton bandana ranged from <5% to 49% [34, 52, 53]. Many of the above studies, with the exception of folded bandana, produced results that are comparable to the cloth mask performance described here. However, it is acknowledged that 3 commercially-available 100% cotton face-coverings, a two-layer bandana, a one-layer mask, and a three-layer mask, tested using a portacount in all particles mode (methodology similar to the present study) produced fitted filtration efficiencies of 0%, 10% and 0%, respectively [34]. Regarding the medical mask control, our testing results (FFE of 55.3±2.1%) were within the range of medical mask fit tests reported in the literature, especially those that tested medical masks with elastic ear loops as fasteners. Generally, medical masks with tie fasteners perform much better than those with ear loops [52]. Medical masks with ties had FFEs from 71.5 to 90% [24, 26, 34, 52], whereas medical masks with ear loops commonly exhibit FFEs between 38 and 63% [52–54] consistent with this study’s findings.

### Mask fit verses material filtration capacity

To evaluate the effect of mask leakage, a mask fitter or a mask overwrap, can be employed in conjunction with quantitative fit testing [14, 26, 28, 39]. It is well recognized that a poorly fitted mask will be unprotective regardless of how efficient the material it is made from is at removing aerosols [13, 22, 24, 26, 39–42, 46]. In the present research, the surgical mask with mask fitter installed had an FFE of 90±0.8% that exceeded its performance by nearly double over its normal use. A similar increase in surgical mask performance with mask fitter in place was reported by Brooks et al. [14]. In contrast, the fabric mask tested with a mask fitter showed little to no performance enhancement. This indicates that the tested fabric mask was operating near its maximum potential filtration efficiency and that its fit was close to ideal. In the tested cloth mask design, fabric ties were used in conjunction with mask channels at the sides of the mask. The ties are able to slide freely through the channels enabling the wearer to fit the mask securely to the face and to bunch the mask sides tightly to the cheeks during mask tying, resulting in a similar effect to the knot and tuck method used to improve surgical mask fit [14, 44]. Mueller and Fernandez [39] tested several versions of cloth masks with and without a mask fitter (the above authors used a nylon overwrap to tightly fix the mask to the wearer’s face). Restricting the reporting of their test results to 100% cotton masks without filters; one 2-ply mask with ear loops had an FFE of 28.2% without a fitter that improved to 73.2% with a nylon overlap. Another 2-ply cotton duck mask with ties showed similar FFEs of 72.5% and 78.5% with and without the fitter while a commercial 2-ply cotton twill mask with ties had relatively similar FFEs of 45 and 66.9% with and without fitter, respectively. These results imply that fabric masks, at least those fastened with ties, can perform closer to their theoretical maximum filtration, whereas medical masks with ear loops, being made of material with excellent filtration properties, perform less well than expected because of edge leak, which is largely corrected by the use of a fitter.

Apart from masks made from bandana, all of the masks and cotton materials tested, have the potential to generate masks with FFE’s equivalent to a 3-ply level 1 medical mask with ear loops in the submicron particle range tested. This has important implications to mask mandates as some jurisdictions and institutions now require use of medical masks and exclude the use of cloth face coverings based on improved filtration performance of commercial medical masks made from synthetic non-woven materials [12]. However, the vast majority of medical masks in use by the public are those that are fastened by elastic ear loops rather than ties and use of mask fitters over medical masks remains rare outside of healthcare settings. Therefore, in terms of protection of the wearer, the expected higher performance of medical masks owing to their potential filtration capability may not be realized in the real world. These results reaffirm that loose fitting medical mask performance can be substantially improved by applying a mask fitter or brace to improve the seal of the mask and generate fitted filtration efficiencies closer to their material filtration capability.

The difference in leakage between the cloth and medical masks also implies a difference in mask performance not captured by the quantitative fit test. Quantitative fit tests measure filtration of particles in the 0.02–1 μm range which include the most penetrating particles: these are the most difficult particles for cloth masks to remove [10]. Alternatively, most cloth fabrics are expected to have much higher filtration efficiencies for larger particles exceeding 5 μm [55] which represent a large fraction of particles generated during coughing and sneezing [18]. Thus, for larger particles, the inward filtration of cloth masks with ties is expected to be higher than that of medical masks with ear loops simply because the edge leak of medical masks admits a large amount of completely unfiltered air. It is anticipated that the same benefits of higher overall mask performance for protection of the wearer across the broad range of particles would also be realized in the case of mask use for source control since inward and outward mask protectiveness tend to be correlated with one another [34]. However, it is recognized that, like medical masks, the vast majority of cloth masks worn by the public also use elasticized ear loops instead of ties as fasteners and we do not yet know to what extent the filtration performance is affected by the overall fit of the mask versus the method of head attachment. In the selected mask design, the Essex Pleated mask using fabric WP045 with elastic ear loops dropped its performance by a small amount (FFE of 48.5% ± 1.1; n = 31 measures; data generated across 5 different mask testers for publication in preparation of a different study), ear loops vs overhead ties might not result in large FFE differences, for this particular cloth mask design. However, the elastic ear loop material used in the Essex pleated cloth mask adopted in this study was thicker and more robust than those typical of disposable medical masks. However, to achieve the maximum benefits of mask performance, additional research to evaluate cloth mask fit across mask designs and head attachment mechanisms should be pursued. Finally, adding more than 2 plies of fabric to the cloth masks would likely increase their filtration capability beyond what was observed in the present study [39].

### Breathability of characterized cotton fabrics

Breathability was measured for 16 selected fabrics via differential pressure testing followed by calibration of an optimized model (Eq 3) to estimate breathability for all 52 cotton fabrics. Pore diameter, fabric weight and pore shape were significant predictors identified by the optimized model. Other studies demonstrated that fabric breathability is positively related to porosity, negatively associated with the tightness of the weave and higher for knits compared to woven fabrics [4]. In the present work there was a small correlation between material porosity and pressure differential but this effect was weak compared to other fabric characteristics and this predictor was ultimately removed during model optimization. Tightness of weave is related to pore size and consistent with the strong negative coefficient associated with pore diameter in Eq 3. Fabric weight also contributed to increasing breathing resistance but has the potential to be differentially influenced between knits vs woven fabric. Davies et al. [24] remarked that tea towel had low breathability compared to T-shirt material but our testing results showed comparable results between samples from both fabric categories. Rogak et al. [48] observed extremely high pressure drops for a down proof 100% cotton ticking, making it unsuitable for masks, and also high pressure drops for a characterized batik fabric. The latter is consistent with our observations whereby 4/5 batiks were predicted to greatly exceeded the medical mask guideline. Other fabric types including bedsheets and home décor were commonly predicted to exceed the medical mask differential pressure limit. However, just as many samples from these same cotton consumer categories had predicted differential pressures much lower than the guideline. Therefore, fabrics from these consumer categories should not be completely ruled out for use in mask making, but there is a greater likelihood of obtaining a material from these categories with poor breathability. All other fabric categories had lower mean pressure differentials that were less than the Level 1 guidance and thus breathability is not expected to be a major limitation to the performance of constructed 2-ply cotton masks. However, breathability will drop further as more layers are added, i.e., 3- or 4-ply mask designs. Masks with low breathability can result in greater leakage during exhalation, coughing or sneezing [4] and contribute to discomfort that leads to lower mask compliance [9, 46]. Thus, some care should be taken when constructing multi-layered (> 2-ply) masks from materials with pressure differentials approaching the breathability thresholds.

### Selecting cotton fabrics for cloth mask construction

Since the start of the pandemic and general recommendations for public use of face coverings, there has been an unprecedented growth of published literature exploring fabric aerosol filtration efficiencies, novel methods for characterizing fabrics as potential source control barriers, and expansion of quantitative fit testing to include medical, consumer grade and home-sewn fabric masks. While this increased attention has contributed to an expanding database on materials characterization of great value towards ‘last resort’ mask research, many studies have remained largely exploratory in nature. A common critique of the current state of the literature is the haphazard selection of materials used for fabric testing and lack of fabric descriptions useful to consumers and home sewists who wish to identify the most appropriate materials that can be used for home mask construction [23, 41, 42, 48]. Beyond being able to identify a high performing fabric, a second issue relates to the local availability of such fabrics to consumers especially under potential supply and global trade interruptions that contribute to the same shortages of commercial PPE. Indeed, while synthetic materials often show much better filtration performance than many natural fabrics [22, 42, 56], some of these materials, such as polypropylene, may be difficult to identify, source and secure for the home sewist and non-commercial mask making organizations. This is especially the case for individuals and groups in low- and middle-income countries, those whose only source of materials to work with are those which can be found at their residence, or situations involving the purchase of re-sale and used items that may have had consumer label information removed.

In the present work, we focused on cotton materials which are recommended by the World Health Organization and U.S. CDC as one type of material that can be used in the production of home-sewn fabric masks. Cotton is the most common type of fabric produced globally [35] but this material can vary substantially in its aerosol filtration capacities [11, 42]. By focusing exclusively on cottons, we were able to replicate a number of samples within consumer categories of intended fabric use or in common cotton commercial products (e.g., bandanas, T-shirts, bed sheets and tea towels) that can be easily recognized by consumers and home sewists and are likely to be locally available. In addition, by including only one type of natural fabric, we were able to develop models of mask performance and material breathability based on microscopic characteristics that was not confounded by different material types contributing to different mechanisms of aerosol filtration. This differs from many studies that characterize a mixture of natural and synthetic materials, some of which possess electret properties and differences in hydrophobic/hydrophilic characteristics. While characterization of mixed fabrics has benefits for finding an optimal filtration solution among the fabrics tested, often there is insufficient replication of any single fabric type to develop predictive statistical models based on fabric characteristics as was accomplished in the present work.

Another important element to consider but rarely discussed is the suitability of different fabrics for constructing masks using standard sewing equipment used by home sewists. In the present study, despite tea towels generating among the best observed and predicted mask FFEs, this material was more difficult to construct masks out of. This is particularly problematic for mask designs that use pleats. One of the tea towels selected for mask construction (WP029) had the highest fabric thickness (344.5 g/m^2^) but could not be fashioned into a mask owing to the inability to sew across multiple layers of the fabric along folded seams. The sewist was able to construct a mask from another tea towel (WP028; 234.5 g/m^2^) but also noted great difficulties in sewing this mask. This suggests that despite fabric weight improving filtration performance, there is an upper limit to fabric weight (<250 g/m^2^) that is practical for homemade mask construction. The authors further note that the tea towel mask was perceptively much heavier to wear, and its pleats did not hold their shape as well compared to other masks tested. Thermal properties or subjective assessments of the masks by wearers were not included in this study; however, we think it plausible that a thick mask may be uncomfortably warm. Thus, even though this material was considered superior for its potential filtration capability, it produced a less desirable mask that may detract from its use and lead to lower compliance and/or more frequent mask adjustments that counteract its somewhat better performance in quantitative fit tests. The only other material that had a negative construction comment was a T-shirt (WP009) where the needle had a difficulty catching the knit loops and the sewist voiced her concern that this may have produced more gaps or holes along the mask seams. One of the bandanas (WP003) used for mask making was smaller in size making alignment of the print pattern of the face fabric difficult, however, this was considered an aesthetic issue rather than a functional one. Davies et al. [24] provided similar commentary on vacuum filter material which had the best filtration capacity of materials tested in their study but proved too stiff to incorporate into a mask.

Finally, studies on material breathability highlighted that some of the tested materials are less suitable for constructing cloth masks due to their low breathability. Cottons purchased as high-quality batik fabrics have a significant probability of having worse breathability than that stipulated for medical masks and therefore this material should be avoided for the construction of cloth masks. Other materials such as bed sheet and home décor exhibited high variation in breathability and although mean breathability of fabrics from these categories did not statistically exceed the level 1 mask guidance value, individual samples did frequently exceed the breathability criteria. Thus, more care should be taken when selecting these fabric types for cloth mask construction. At the very least, when using these materials, prototype masks should be donned and carefully evaluated for user discomfort with respect to ease of breathing while wearing the mask in the intended way.

Across the original nine consumer categories evaluated for prospective 2-ply cotton mask construction, 3 consumer categories are excluded due to potential issues of either poor filtration, poor breathability or difficulty with sewing. These include bandana (poor filtration), tea towel (difficult to sew due to material thickness) and high-quality batiks (poor breathability). We also note high variability in the breathing performance of some fabrics including home décor and very high thread count bedsheets that warrants additional caution in the use of these materials for mask construction. The remaining cotton consumer categories: T-shirts, fashion fabrics, mass market quilting cotton and high-quality quilting cotton materials on average met sewability, breathability and filtration requirements to yield cloth masks that can achieve comparable performance to that of a level 1 medical mask donned with ear loops provided the mask user can achieve a near optimal fit.

### Study limitations

Despite characterizing 52 different cotton materials, the number of cottons examined in the present research still represents only a very small fraction of the total diversity of cotton fabrics available to consumers and it remains unknown if Eqs 2 and 3 would retain their same degree of predictive power across a much larger range of cotton fabric types. Notably, this study did not include cotton flannels which tend to perform well compared to woven cottons [22, 38, 45, 56] but was not originally considered because of it was thought to be too thermally insulating to be comfortable. Eq 2 is also unlikely to be applicable to fabrics that do not consist of 100% cotton because other material types, especially hydrophobic synthetic materials, will retain particles by different entrainment mechanisms including electret and triboelectric properties [38, 43]. Hydrophobic materials also generate higher surface tensions to droplets along their pore perimeters, thus increasing the energy required for droplets to squeeze through them and resulting in higher filtration performance compared to hydrophilic fabrics with the same pore dimensions [43]. This would likely lead to a larger magnitude of the pore diameter coefficient if Eq 2 were calibrated using hydrophobic fabrics. The hydrophilic disadvantage of cottons may be partially compensated by using post-production fabric treatments that increase water resistance. Crilley et al. [22] demonstrated that treatment of flannel with a water repellent product improved its filtration performance by 8.1% over the same untreated fabric. Despite some of the above disadvantages of cotton fabrics, cottons are still routinely recommended for mask construction [10, 20, 22] or are recommended as one of multiple layers incorporated into multi-fabric mask designs [4, 19, 43]. Therefore, the results from the present study have applicability for selecting appropriate cottons used in masks consisting of 100% cotton or masks that include cotton as one of multiple fabric layers in the mask design.

A stated advantage of cloth masks over commercial PPE is their greater sustainability due to their ability to be cleaned and sterilized through normal laundering facilities. However, this study did not examine whether attributes such as mask fitted filtration efficiency and breathability change across multiple washing cycles. Reutman et al. [56] compared aerosol filtration performance (0.04–3.2 μm size range) across a 3-layer fabric combination selected for use in a homemade mask prototype across 10 washing cycles. The layers included polycotton as the outer and inner fabrics and a mid-layer of polypropylene. Aerosol retention reportedly decreased from 78.1–85.8% to 55.9–69.3% across two face velocities (30 L/min and 85 L/min) following the 10 cycles washing. The authors reported no change in material breathability related to laundering. It is not known if the change in filtration performance described above relates to changes in polypropylene known to be sensitive to heat or the polycotton fabrics used in the mask construction. Bhattacharjee et al. [13] subjected cottons to two machine washing cycles at 60°C and observed a slight increase in aerosol filtration performance interpreted to be due to fabric shrinkage over its unwashed state and tightening of the weave related to this initial treatment. The authors noted that degradation in performance may be observed after subsequent cumulative washing cycles. Hao et al. [41] subjected eight fabrics, including a cotton, to 1, 5 and 10 machine washing cycles and drying. Similar to Bhattacharjee et al. above, Hao et al., observed an increase in material filtration performance of the tested cotton after 1 washing and this increase in performance relative to unwashed material was retained up to 10 washing cycles (the maximum cycles tested). However, further research to investigate cloth mask performance and end-of-life washability across consumer cotton types should be performed to better understand constraints to mask re-use under normal laundering activities.

Finally, the present research was limited to testing a single 2-ply mask design on two mask wearers, with all of the material mask comparisons being tested on a single mask wearer. These limitations were purposely imposed in order to maximize inferences about differences in mask performance to the materials being tested rather than separate evaluations of mask design or fit across different mask users. However, home sewn-masks vary tremendously in mask design, number of fabric layers, fabric type, presence of filter pockets and filters, fastener type and other attributes [13, 22, 57]. The U.S. CDC currently recommends masks with two or three layers of washable breathable fabric that include cotton and cotton blends. World Health organization recommends cloth masks be made of 3 layers, an inner layer of hydrophilic material such as cotton, a middle layer with strong filtration properties such as polypropylene and an outer layer of hydrophobic material such as polyester or polyester/cotton blend. The design features of 3-ply blended-material masks recommended above offer a combination of properties of water repellency for out-facing fabrics, electret properties which increase charged particle retention at the middle layer [4, 13, 45] and liquid interception on the inner fabric that work together to achieve both inward and outward protection. However, it must be emphasized that superior materials do not always generate a superior mask if the materials used, relating to material drape/pliability, and mask design, relating to type of head attachments, do not contribute to a good fitting mask, as was demonstrated in our work by the 3-ply medical masks on ear loops. Therefore, mask prototypes need to be tested and carefully evaluated for fit on particpants rather than just relying on theoretical material performance as the primary factor in material selection criteria.

The present study demonstrates that simple 2-ply cotton cloth masks of selected materials from common consumer categories (T-shirts, fashion fabrics, mass market quilting cotton and high-quality quilting cotton materials) meet sewability, breathability and filtration requirements to achieve aerosol protective properties equivalent to level 1 medical masks owing to superior fitting characteristics. Cloth masks offer more sustainable, low cost alternatives to medical masks because they can be sewn by most individuals using materials already present at their residence or readily sourced from local markets that are less subject to supply interruptions as is the case with single-use disposable medical masks and for some types of recommended cloth mask making materials such as spun-bound non-woven polypropylene. For volunteer organizations engaged in cloth mask making for donation purposes, and in low- and middle-income countries, there may also be cost-advantages to adopting a 2-ply over a 3-ply mask design since adding an additional 3^rd^ layer will result in 30% lower mask production output for the same amount of materials. Mask output in this setting, where materials are donated, or where funds for material purchase are limited, can be optimized when a number of different broad classes of materials are known to be suitable, as shown in this work. In designing commercial masks, third layers and hybrid composition should be considered and tested. The cost-benefits of improved performance of a 3-ply all-cotton or hybrid-layer design should be weighed carefully against the organization’s aims at maximizing mask production, in the context of a mask-for-all mission.

## Conclusion

This study characterized 52 cotton fabrics categorized into 9 consumer categories based on the intended sewing or consumer goods application. A selection of 16 fabrics were used to construct 2-ply cloth masks of the same design and subjected to quantitative fit testing and material breathability performance. A statistical model was able to explain 81% of the variation in mask fit testing performance and 92% of material breathability based on fabric characteristics of pore diameter (μm), pore shape, and fabric weight (g/m^2^). The models were then used to predict the potential mask performance of all 52 characterized cottons. Model results indicate that cotton fabrics from 8 of 9 consumer categories can produce fabric masks with performance potentials equal to an L1 medical mask with ear loops. The only consumer category that underperformed were masks made from cotton bandanas. Among the top performing cottons were tea towel, home décor, batik quilting cotton, high-quality quilting cotton and bedsheet followed closely by t-shirt, fashion fabrics and mass marketed quilting cottons. Tea towel material had high fabric thickness that contributed to difficulties in constructing masks using conventional home sewing equipment. Poor breathability was also observed for high-quality batik quilting cottons and high variations in breathability were observed for fabrics designated as home décor and some high thread count bed sheets. Consumer categories that predictably exhibited fitted filtration efficiency comparable with that of a medical mask, acceptable breathability, and suitability for construction of a pleated mask were T-shirt, fashion fabric, mass-market quilting cotton, home décor fabric, bed sheets and high-quality quilting cotton. For a single mask made of high-quality quilting cotton, the mask performance with and without a mask fitter was statistically equivalent. This indicates that most of the air entering the mask was filtered by the mask material in the tested mask design and contrasts with the medical mask with ear loops where nearly 50% of air entering the mask was derived from leaks. Thus, even though the cottons tested have poorer overall aerosol filtration efficiency, their ability to remove 0.02–1 μm sized aerosols was similar to the medical mask due to less edge leak, which was most apparent with the medical mask. We predict that well fitted multi-layer cloth masks will provide even greater protection across a larger range of particles emitted by humans because of lower edge leakage. Thus, 2-ply all-cotton masks of recommended consumer-grade materials can provide low-cost, more sustainable alternatives to single-use loose fitting disposable medical masks. Importantly, such masks, over multiple wearings, are more economical for the mask wearer and can be readily made by most home sewists or volunteer organizations using locally-sourced materials to achieve mask-for-all mandates under situations where supply interruptions can interfere with medical-mask procurement or where financial limitations preclude constant replenishment of disposable medical items. Finally, this research supports the use of a mask fitter in conjunction with disposable level 1 medical masks in order to achieve mask performances more commensurate with their high material filtration capability which exceeds the performance of 2-ply cotton masks with ties evaluated in the present study.

## Supporting information

S1 Table Fabric source, ESEM fabric characteristics and quantitative fit testing of selected cloth masks. (XLSX)

S1 File Essex pleated mask construction instructions. (PDF)

S2 File Photos and ESEM images for each fabric used in testing. (PDF)
