# Supplementary material for: Fitted filtration efficiency and breathability of 2-ply cotton masks: Identification of cotton consumer categories acceptable for home-made cloth mask construction
Source: PLoS One. 2022 Mar 22;17(3):e0264090. doi: 10.1371/journal.pone.0264090 (PMC8939836; doi:10.1371/journal.pone.0264090)
Supplement: S1 File — (PDF) [file pone.0264090.s002.pdf]

Appendix S2. Essex Pleated Mask Construction Instructions

Created 8/30/2021

These instructions were created by Windsor-Essex Sewing Force as part of a WE-SPARK Health Institute and University of Windsor funded grant project. Windsor-Essex Sewing Force would like to thank our collaborators who continue to work with us to create the best homemade mask possible.

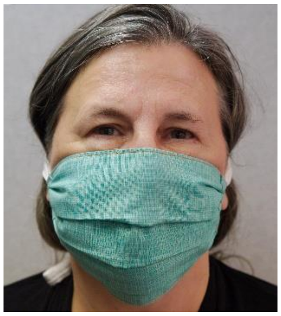

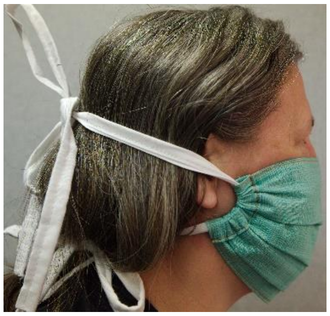

Level of difficulty: Easy
If you aren't a confident sewist or if you've never made masks before, we suggest you start with this mask, the **Essex Pleated Mask**.

This document includes sewing instructions, photos and tips and sewing term explanations for each step.

Instructions	***Photos***	***Tips and sewing term explanations***
Sew fabric and lining together		
1. Cut two pieces of colour contrasting, good quality cotton quilting material 9”x9” (228.6 mm x 228.6 mm) square, keeping the edges along the straight grain. The darker colour will be the fabric of the mask that faces outward, and the lighter colour will be the lining that will go toward the wearer’s face. Cut two more pieces of the fabric 2½”x5” (63.5 mm x 127 mm), keeping the edges along the straight grain of the material. These will be the channels of the mask.	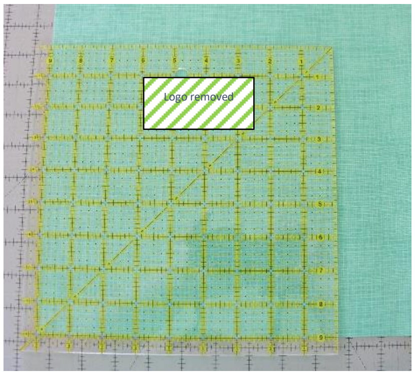 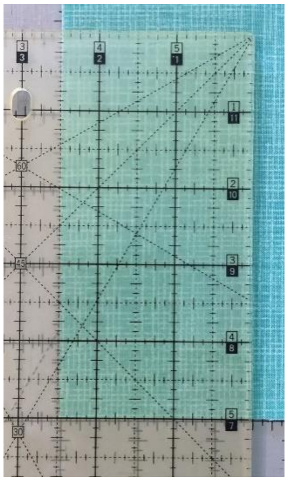	*Grain* – Woven material is composed of two sets of threads that run at right angles to each other. Cutting along the straight grain of the material means that the straight edges of the piece are cut parallel to the woven threads.<br>*Fabric* – The material that will face outward when the mask is complete.<br>*Lining* – The material that will face the wearer’s face when the mask is complete.<br>*Channel* – Or casing. The channels are the tubes of material through which ties or elastic are threaded that the wearer will place around his/her head or ears, respectively, to keep the mask in place.
2. Lay the fabric on the lining, right sides together, matching sides and corners exactly.	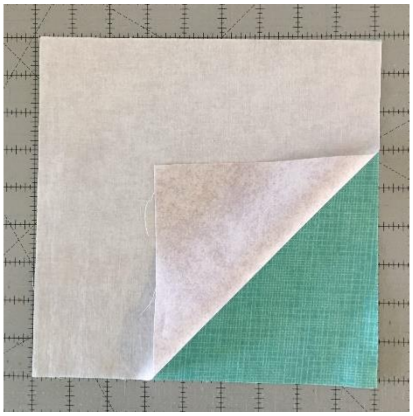	

Instructions	*Photos*	*Tips and sewing term explanations*
3. Stitch the layers together along the top and bottom with a ¼” (6.4 mm) seam allowance.	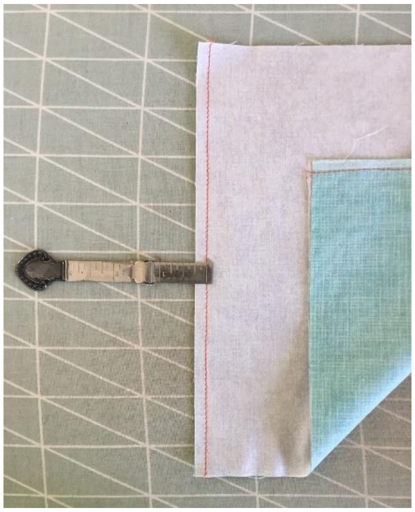	*Seam allowance* – The material between the stitching line and the cut edge.<br><br>The pieces of material are square. Be sure to choose “top” and “bottom” carefully, based on the print design of the fabric. If the design has an “up” and “down”, be sure the “up” is at the top.<br><br>*Backstitch* – Insert the needle three or four stitches from the start of the seam and stitch backwards to the start of the seam before stitching the seam proper. This prevents the seam from coming apart at the start. Backstitching three or four stitches at the end of the seam before cutting the thread also prevents the seam from coming apart at the end.<br><br>Backstitching at the start and end of this seam is *not* necessary.<br><br>When making several masks in assembly line fashion, this seam can be stitched for each mask one after another without breaking the thread in between, similar to quilt piecing. This saves thread and time.

Instructions	*Photos*	*Tips and sewing term explanations*
4. Press the seams open. Turn the layers so that the right sides are out and the wrong sides are together. Press the seams flat.	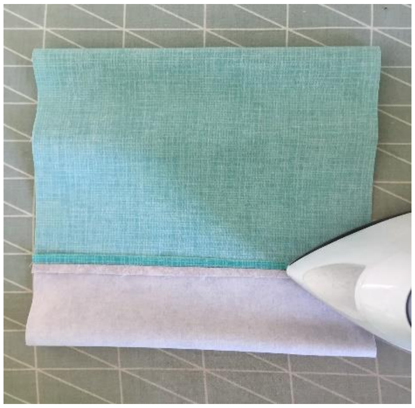<br>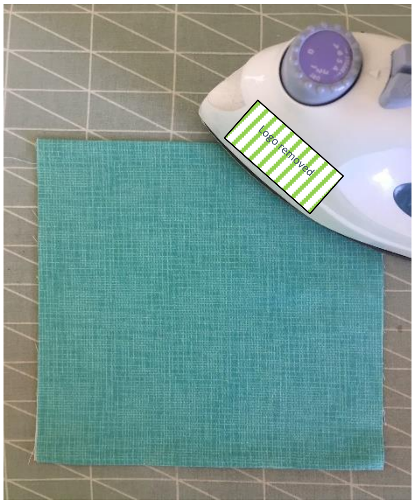 	Pressing the seams open first makes it easier to get them pressed flat after turning. Be sure that the lining does not show along the seamline when the fabric is facing you.
Make pleats		
5. Fold mask in half lengthwise, fabric side out. Press. Open the mask out with the lining side facing you. Bring the bottom long edge to the centre fold just made. Press. Similarly, bring the top long edge to the centre fold just made. Press.	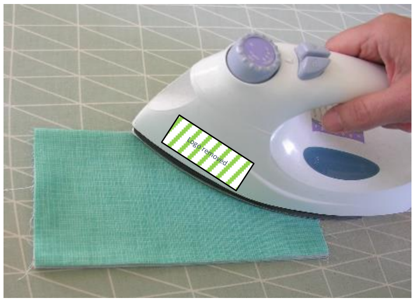<br>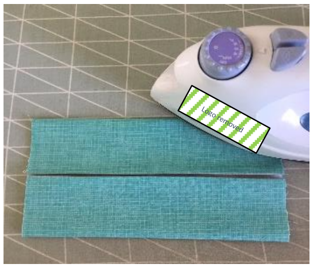	Be careful not to flatten the first centre fold you made as you make the subsequent two folds.

Instructions	*Photos*	*Tips and sewing term explanations*
6. Open the mask again, and place it lining side down, top away from you, on the ironing board. Starting with the bottom fold, make a pleat by bringing the bottom fold toward the bottom of the mask, keeping it parallel to the bottom of the mask. Press. Then bring the centre fold toward the bottom of the mask so it is parallel with the first underside fold you made but not overlapping. Press again. Finally, bring the top fold toward the bottom of the mask so it is parallel with the second underside fold you made but not overlapping. Press one last time. Clip the pleats if you wish. When this step is completed, the sides of the mask should have the same length of 3¾” to 4” (95.3 to 101.6 mm).	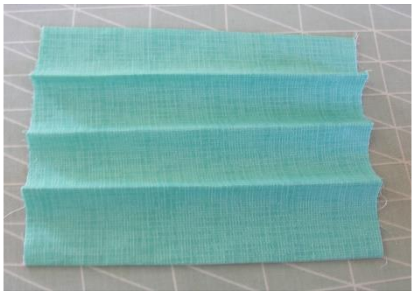 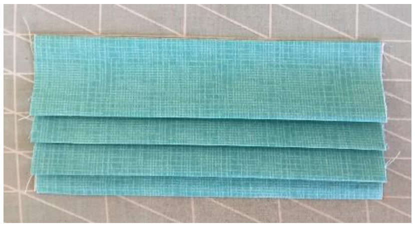 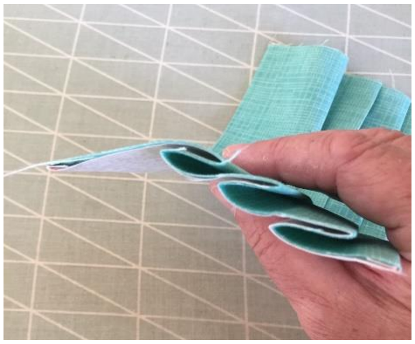 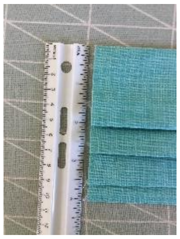 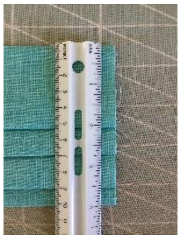 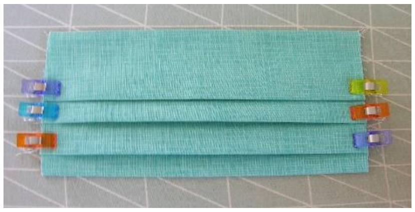	Be careful again not to flatten any of the previously made folds as you make the new ones.<br><br>Clips are preferred to reduce the number of perforations in the fabric. If pins are used, keep the perforations inside the seam allowances.<br><br>Attach the clips perpendicular to the cut edge and parallel to the pleat fold. Make sure the raw edges line up. Care and accuracy is especially required in this step to ensure pleat quality.

Instructions	*Photos*	*Tips and sewing term explanations*
7. Stitch the pleats with a ¼” (6.4 mm) seam. Press flat.	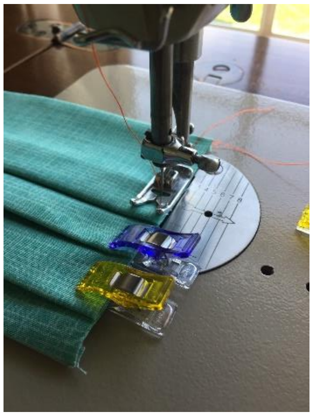 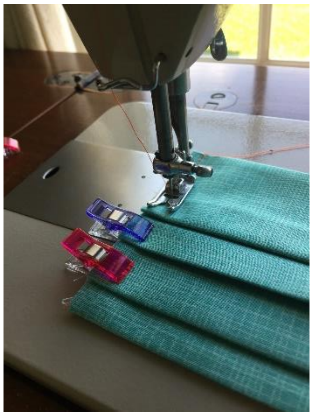 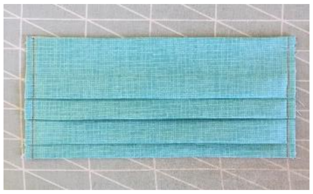	Stitch the pleats from top to bottom to prevent the material from shifting. For one side, you will have the mask positioned to the right of the needle which can be awkward, but the pleat quality will be better.<br><br>Remove the clips as you go.<br><br>Backstitching at the start and end of the seam is not necessary.<br><br>When making several masks in assembly line fashion, this seam can be stitched for each mask one after another without breaking the thread in between, similar to quilt piecing. This saves thread and time.
Add channels		
8. Clip one long edge of the channel to the *lining side* of one side of the mask, right sides together, centering it and matching the raw edges. You should have about 3/8” (9.5 mm) excess at each end. Fold that excess to the fabric side of the mask, keeping the short edges parallel with the top and bottom of the mask, and clip.<br><br>Repeat for the other side.	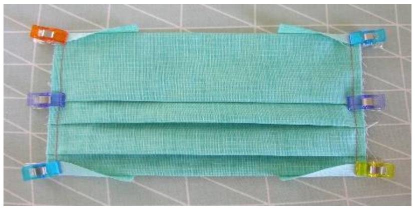	

Instructions	***Photos***	***Tips and sewing term explanations***
9. Stitch with a scant ½” (12.7 mm) seam. Press the channel away from the mask, turning the short edges right side out and pressing them parallel to the top and bottom of the mask.<br><br>Repeat for the other side.	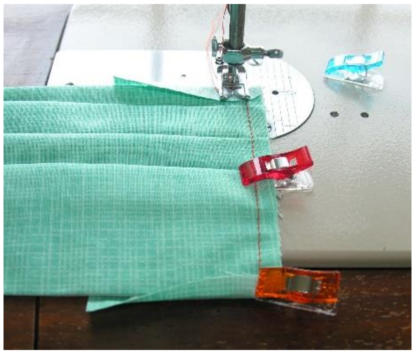<br>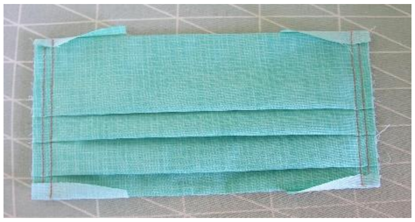<br>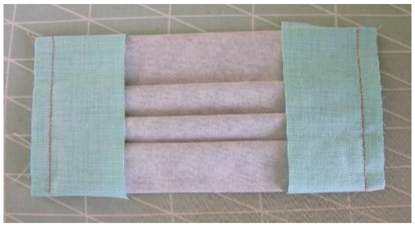<br>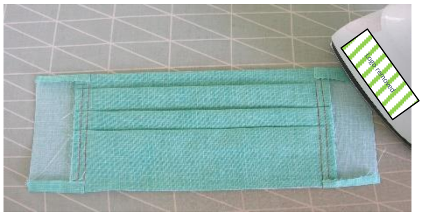	*Scant seam* – Stitch so that the seam allowance is slightly less than the specified measurement.<br><br>Backstitching at the start and end of the seam is not necessary.<br><br>When making several masks in assembly line fashion, this seam can be stitched for each mask one after another without breaking the thread in between, similar to quilt piecing. This saves thread and time.

Instructions	***Photos***	***Tips and sewing term explanations***
10. Flip the mask over so that the fabric is facing you. Topstitch along the entire length of the top and bottom of the mask 1/8" (3.2 mm) from the edges.	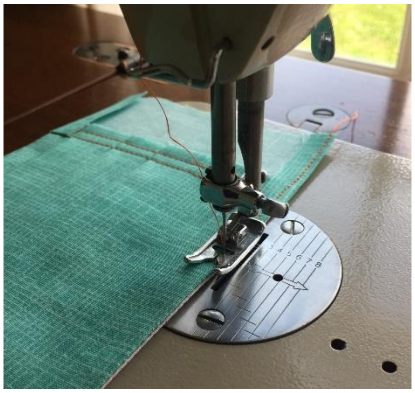<br>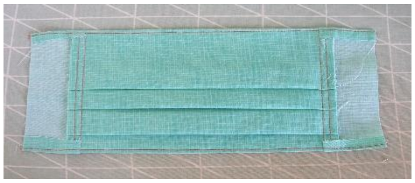	*Topstitch* - make a row of continuous stitches on the top or right side of a garment or other article as a decorative feature.<br><br>Backstitching at the start and end of the seam is not necessary.<br><br>When making several masks in assembly line fashion, this seam can be stitched for each mask one after another without breaking the thread in between, similar to quilt piecing. This saves thread and time.
11. Fold the long raw edges of the channels 3/8" (9.5 mm) from the right side to the wrong side, toward the mask fabric. Press. Fold the folded long edges of the channels over the side edges of the mask so that they barely cover the stitching line you just made in step 9. Press. Check the width of the mask at this point to ensure that it is no more than the required width of 9" (228.6 mm) . If it is too wide, adjust the size of the first fold you made on both sides of the mask until the size is correct. Clip if you wish.	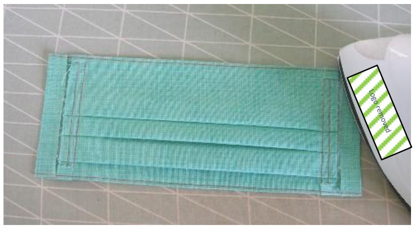<br>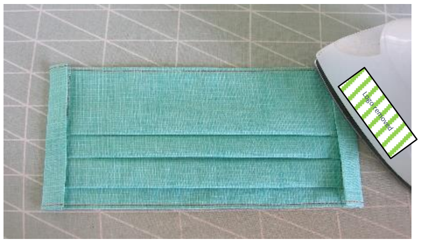	

Instructions	Photos	Tips and sewing term explanations
12. Stitch the channels to the mask as close to the open folded channel edge as possible, no more than 1/8” (3.2 mm).	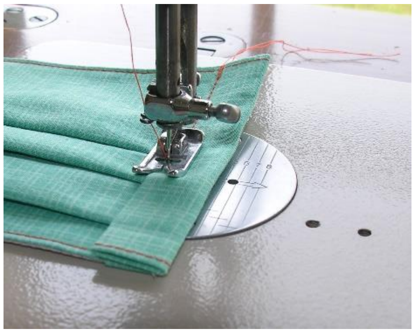 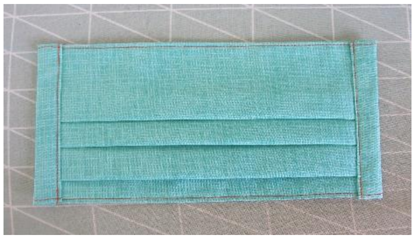	Backstitching at the start and end of the seam *IS* necessary in this step. This reinforces the strength of the seam at a particular wear point.
Fabric ties		

Instructions	***Photos***	***Tips and sewing term explanations***
13. Cut two pieces of fabric 2”x36” (50.8 x 914.4 mm) \, keeping the edges along the straight grain.	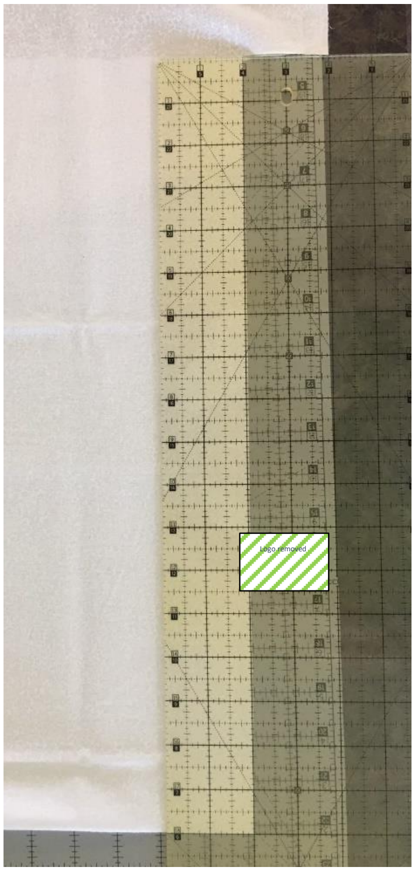	As shown in the photo, if your cutting mat is too small to cut the entire 36” length, fold the fabric in half along the straight grain and cut 18” (457.2 mm).

Instructions	***Photos***	***Tips and sewing term explanations***
14. Press the ties in half lengthwise, wrong sides together. Open the ties out and press ¼” (6.4 mm) of the short edge to the wrong side. Press the long edges of the ties toward the centre fold line. Fold the ties lengthwise in half again to encase the raw edges, including the short ends, and press again.	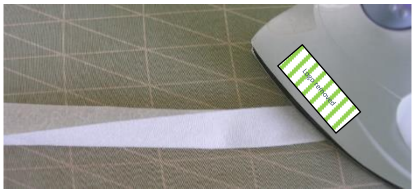 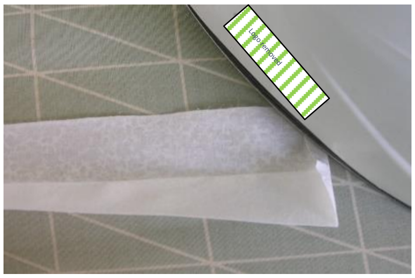 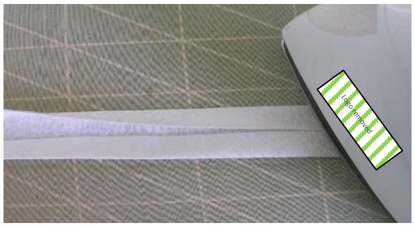 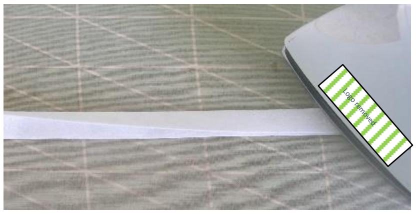	See the video in the “Sewing Room” tab at www.wesf.ca for tips on using a tape maker to make this step easier.
15. Stitch lengthwise along the open edges, making sure to catch all fabric layers.	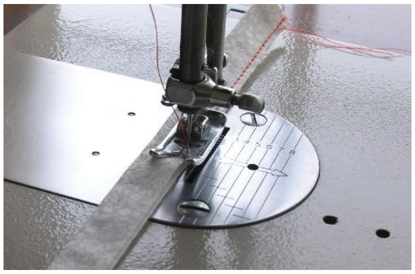	Backstitching at the start and end of the seam *IS* necessary in this step to prevent the ties from coming apart at the ends.

Instructions	*Photos*	*Tips and sewing term explanations*
16. Thread the ties through the channels using a bodkin. Tie with a loose knot so that the ties do not leave the channels.	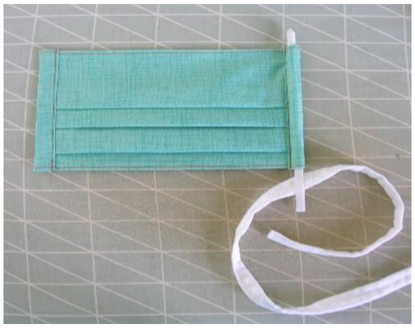 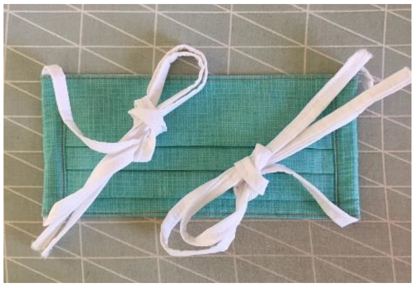	Ensure no channel raw edges are visible after threading the ties through them.
