# Supplementary material for: Fitted filtration efficiency and breathability of 2-ply cotton masks: Identification of cotton consumer categories acceptable for home-made cloth mask construction
Source: PLoS One. 2022 Mar 22;17(3):e0264090. doi: 10.1371/journal.pone.0264090 (PMC8939836; doi:10.1371/journal.pone.0264090)

Supplementary Information Fabric Photos and ESEM Images

WP001
Bandana

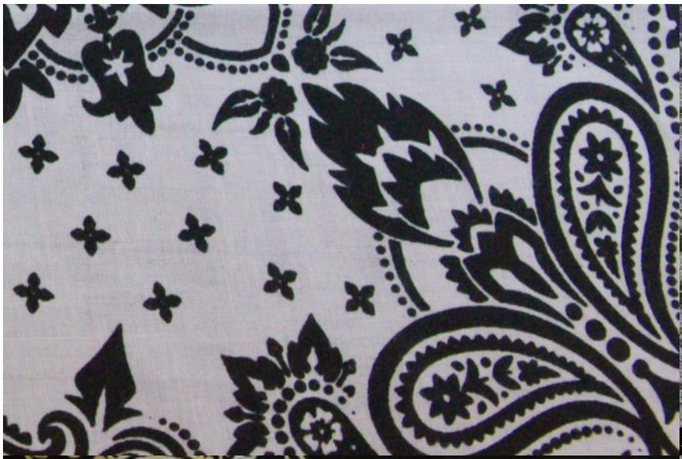

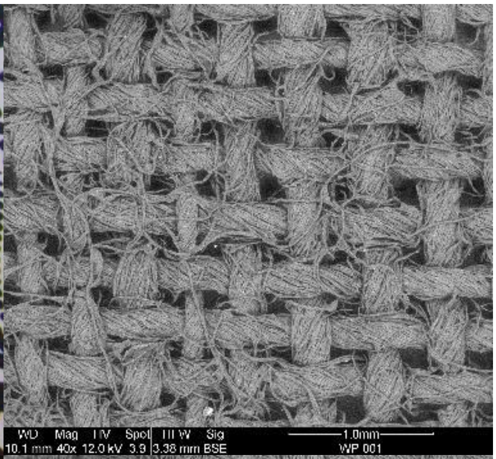

WP002
Bandana

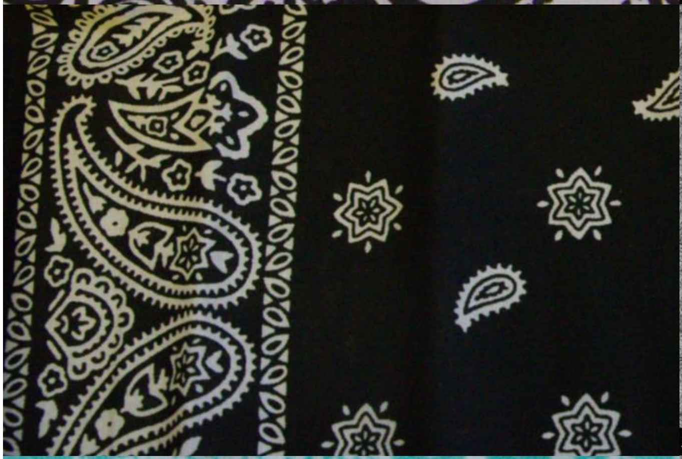

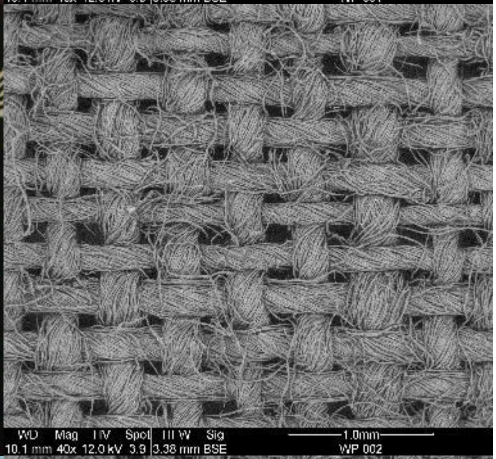

WP003
Bandana

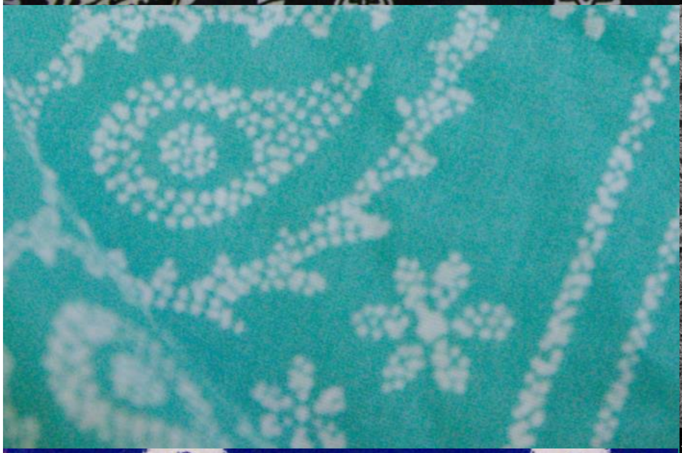

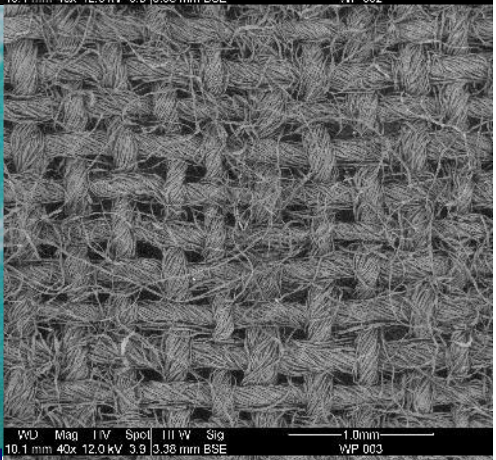

WP004
Bandanda

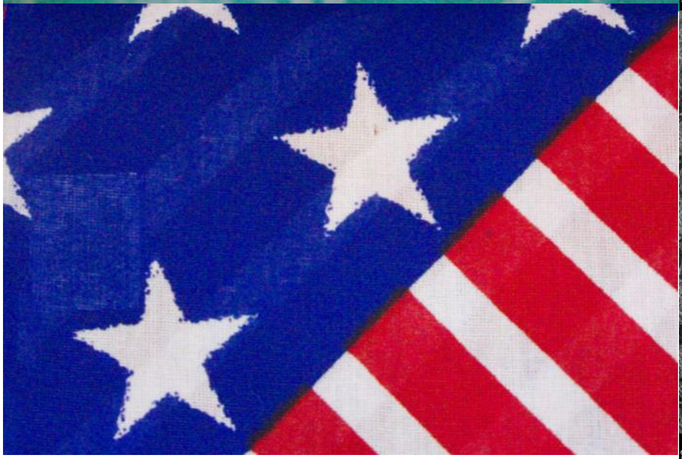

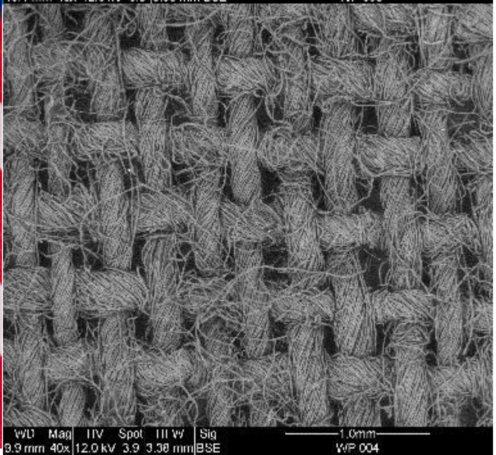

Supplementary Information Fabric Photos and ESEM Images

WP005
Bandana

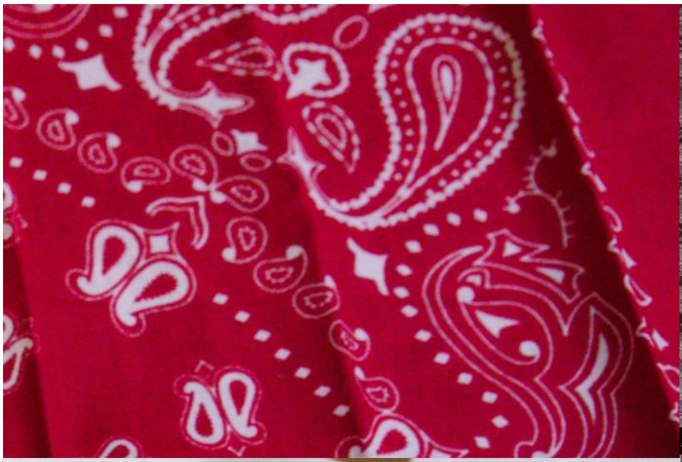

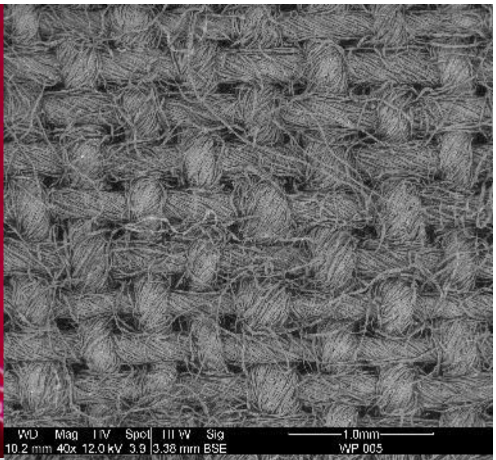

WP006
t-shirt

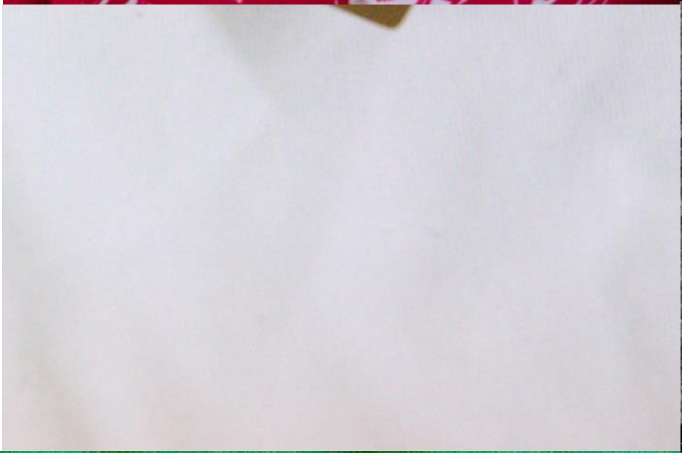

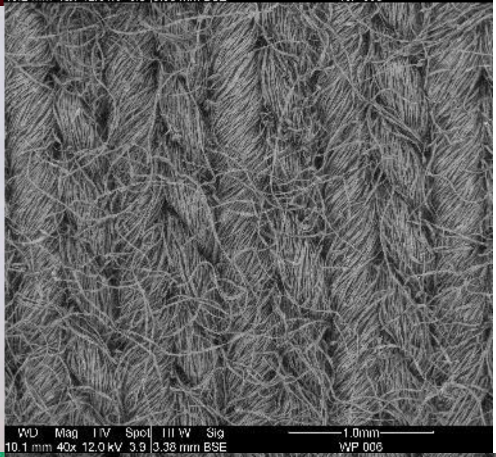

WP007
t-shirt

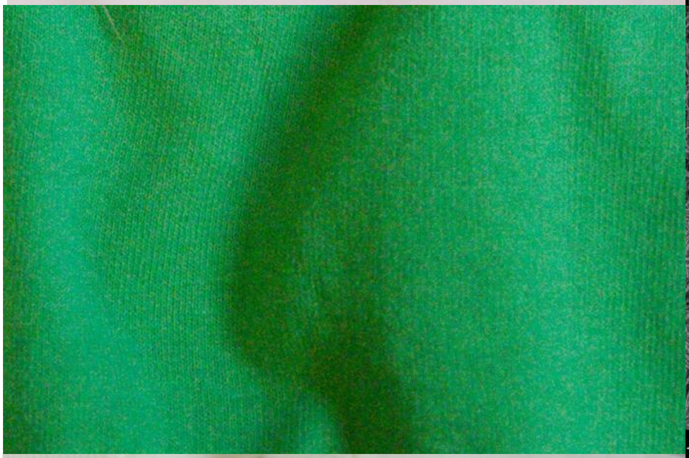

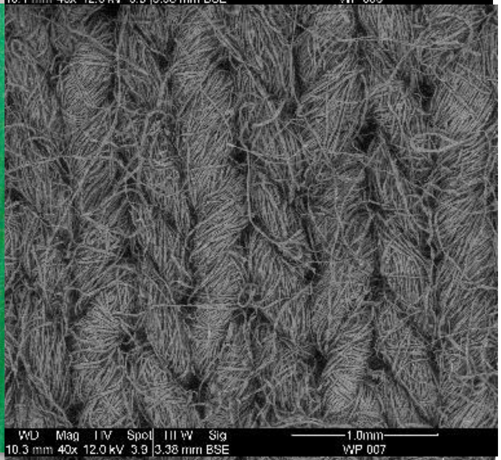

WP008
t-shirt

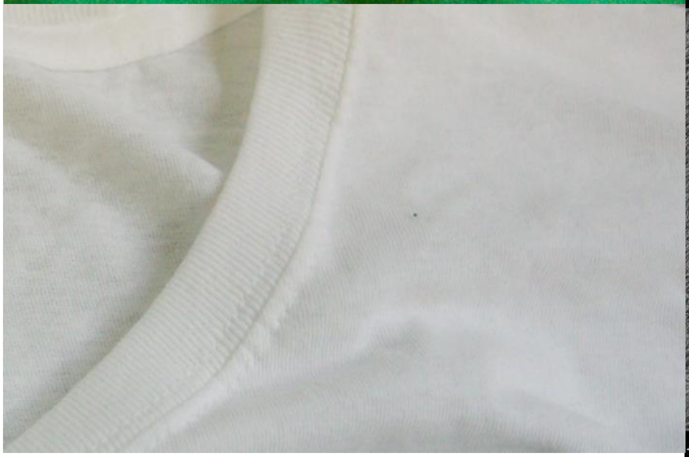

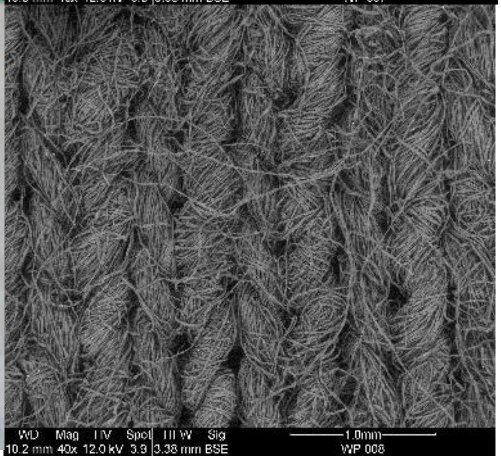

Supplementary Information Fabric Photos and ESEM Images

WP009
t-shirt

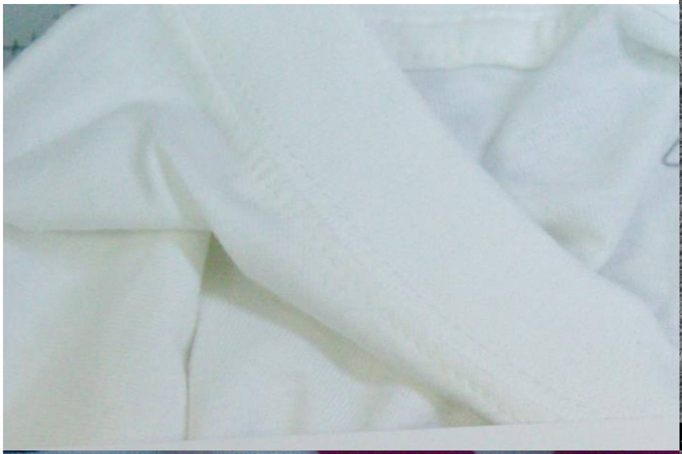

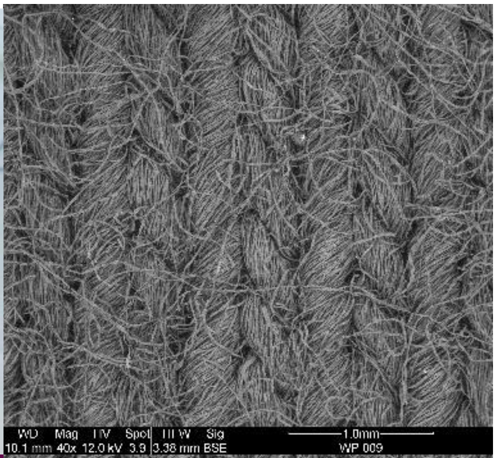

WP010
t-shirt

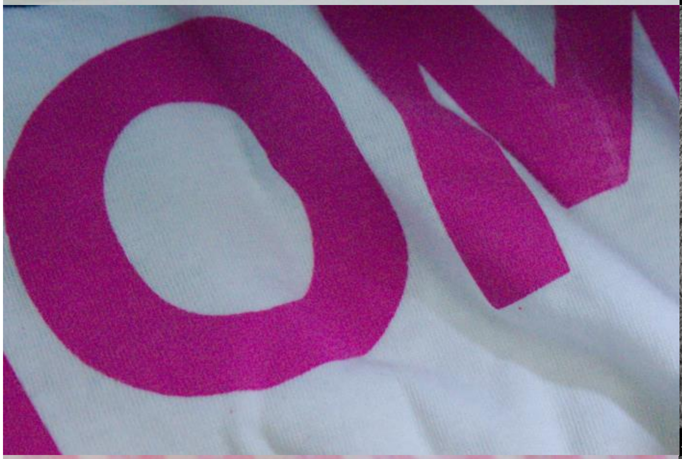

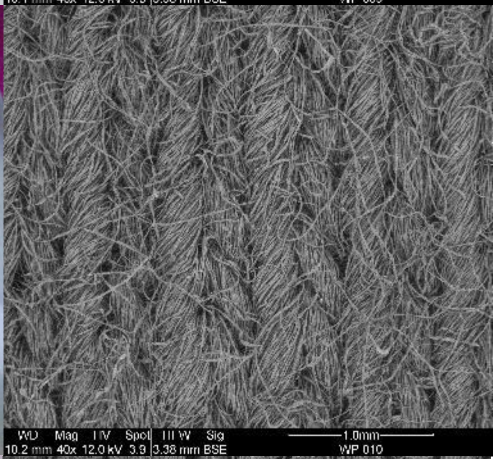

WP011
Fashion
Fabric

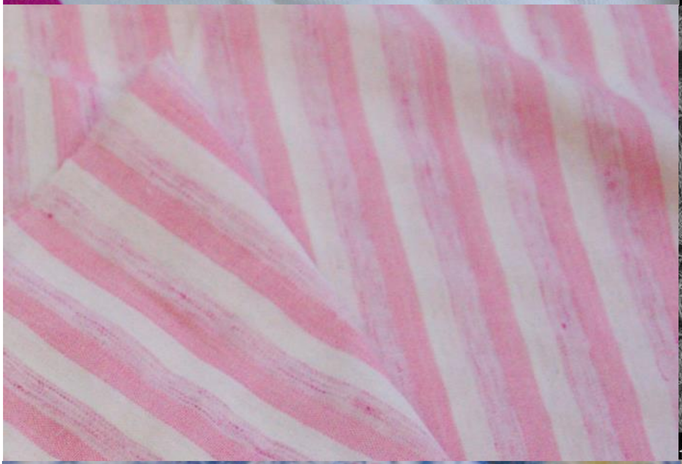

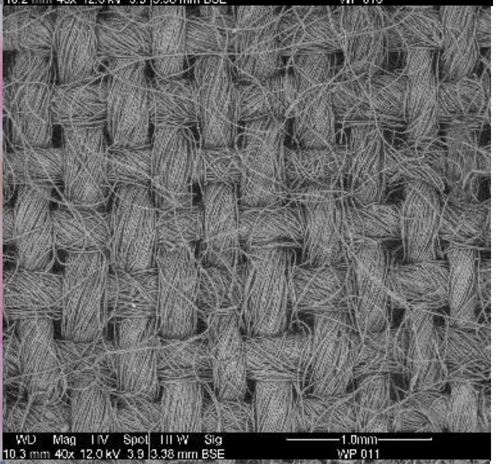

WP012
Fashion
Fabric

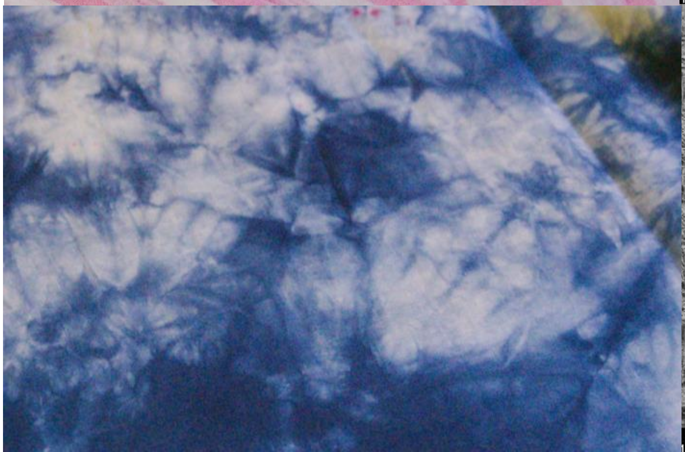

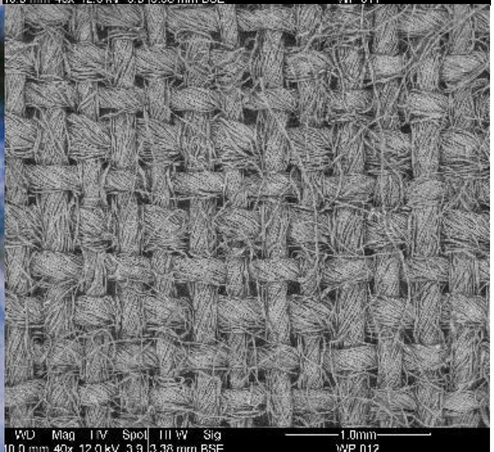

Supplementary Information Fabric Photos and ESEM Images

WP013
Fashion
Fabric

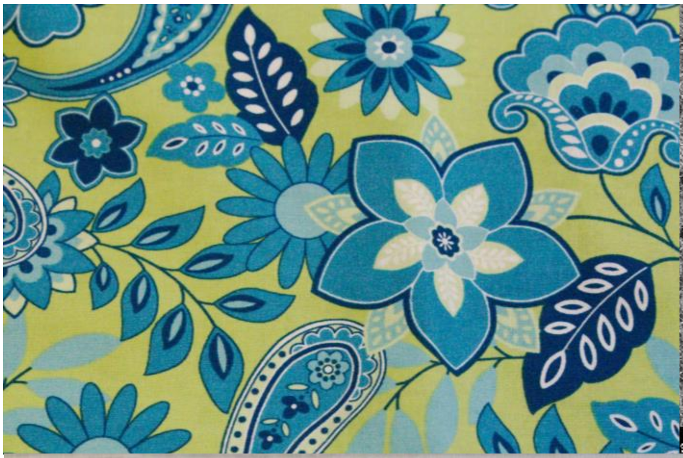

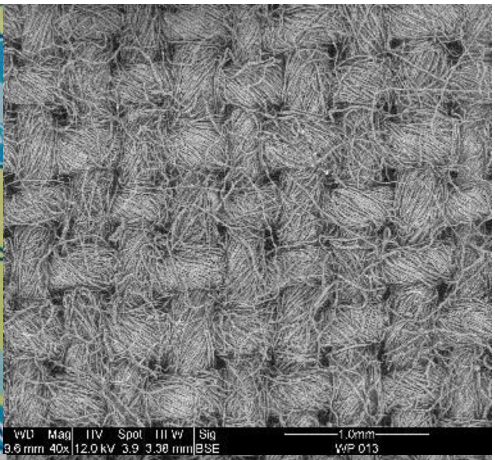

WP014
Fashion
Fabric

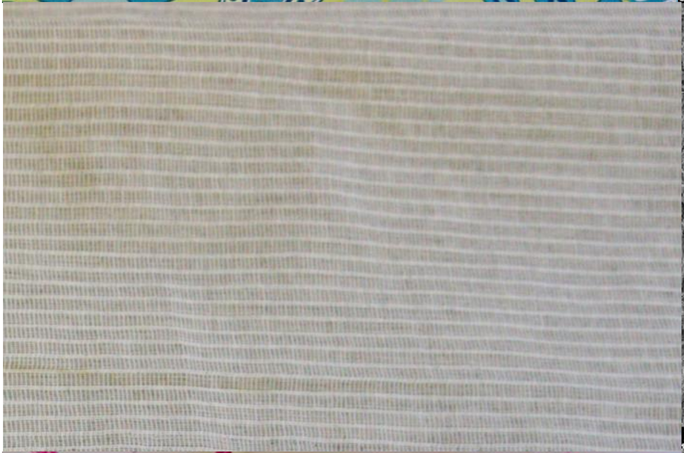

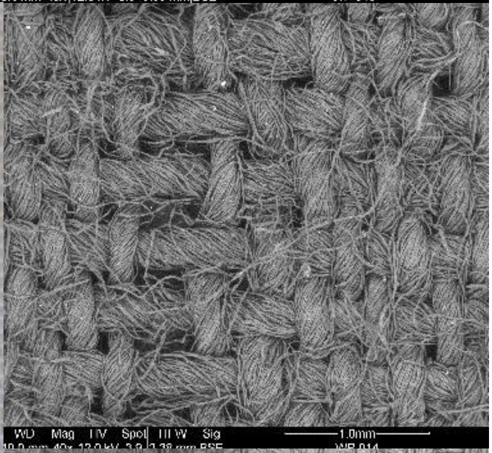

WP015
Fashion
Fabric

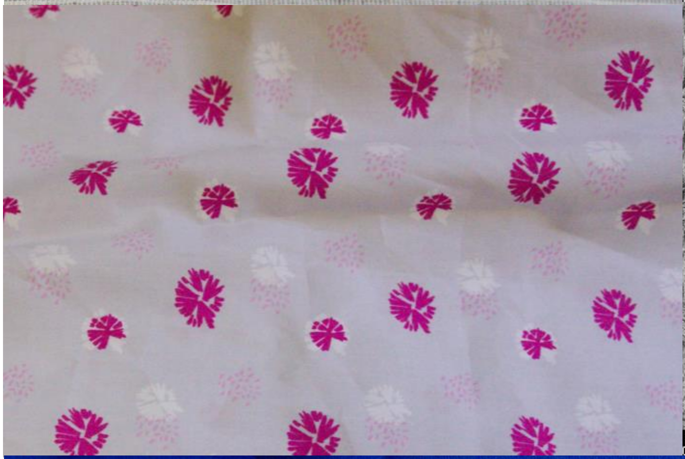

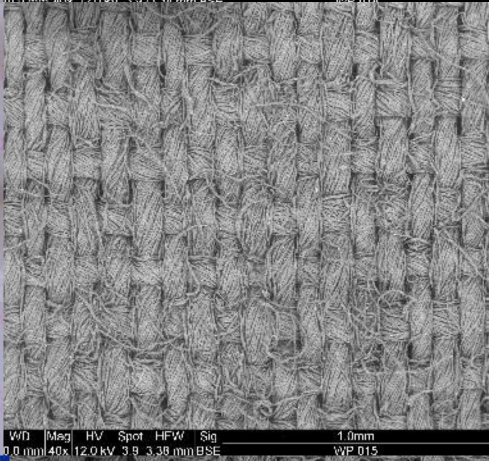

WP016
Mass Market
Quilting
Cotton

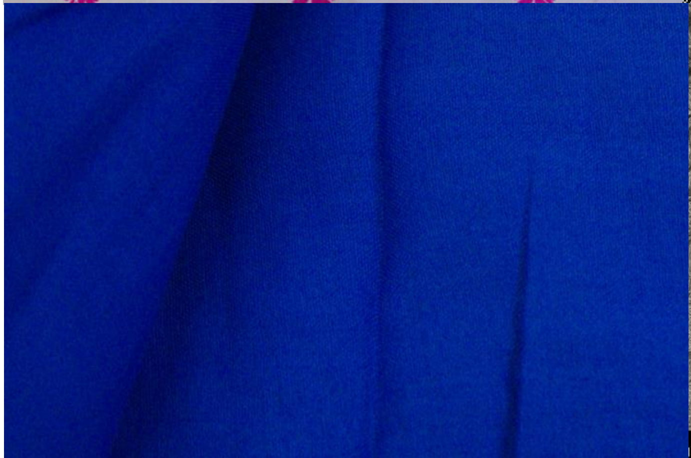

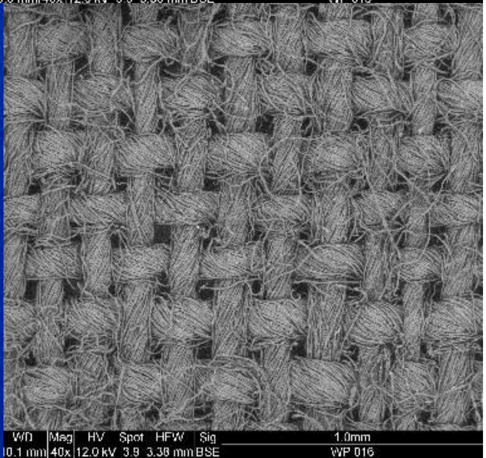

Supplementary Information Fabric Photos and ESEM Images

WP017
Mass Market
Quilting
Cotton

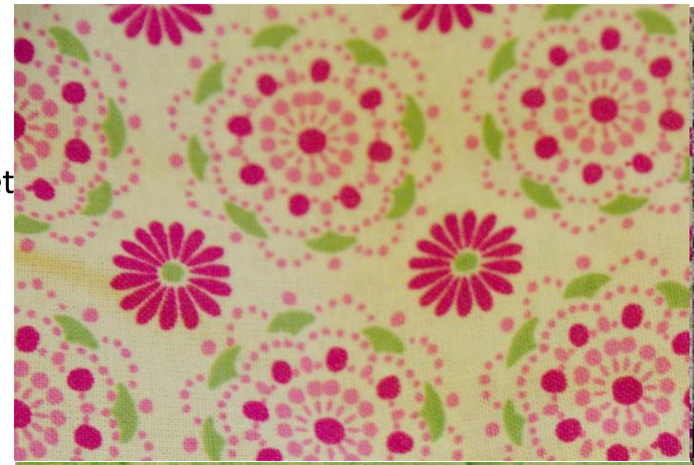

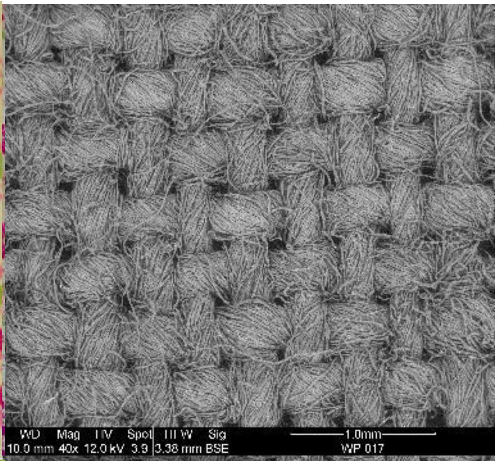

WP018
Mass Market
Quilting
Cotton

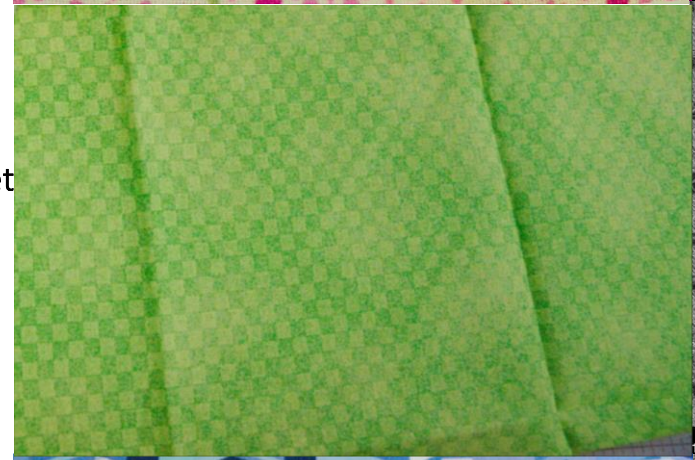

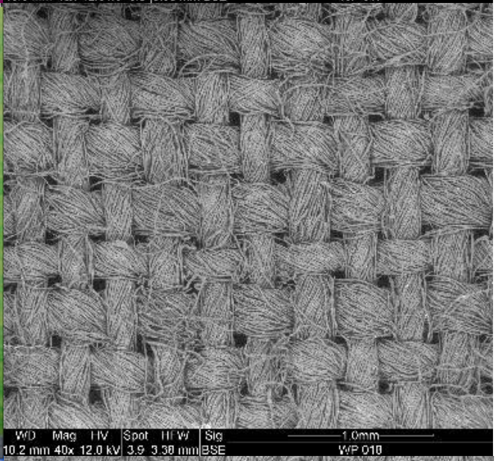

WP020
Mass Market
Quilting
Cotton

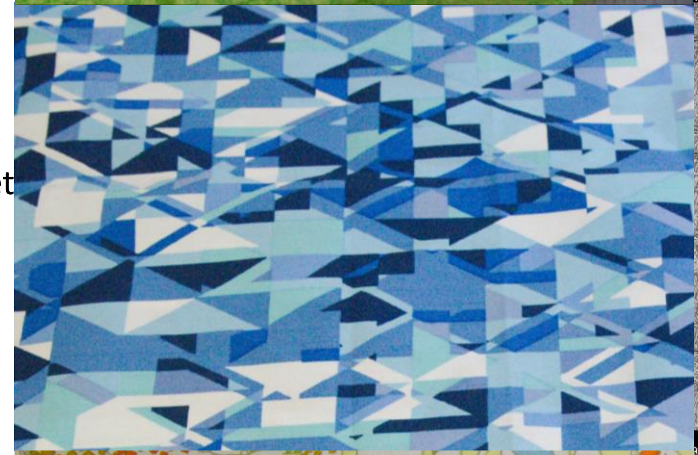

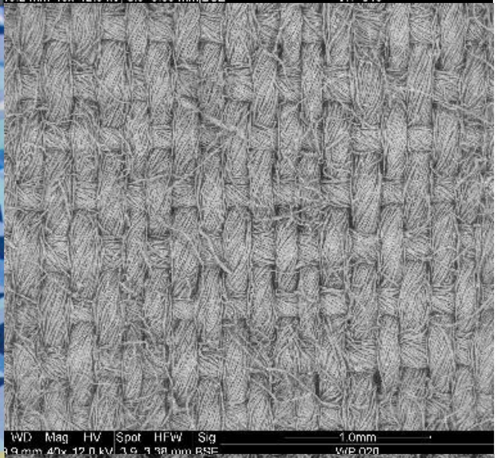

WP021
Mass Market
Quilting
Cotton

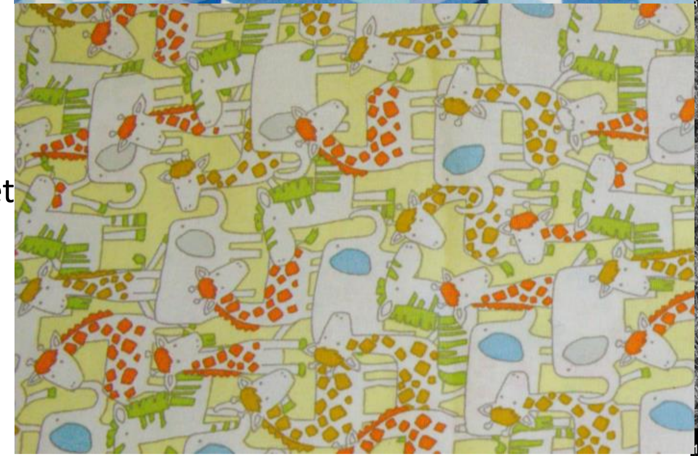

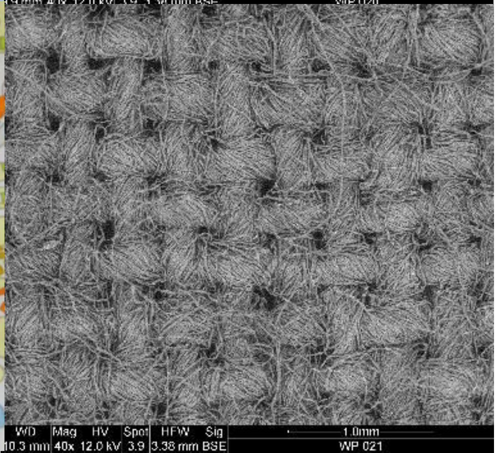

Supplementary Information Fabric Photos and ESEM Images

WP022
Mass Market
Quilting
Cotton

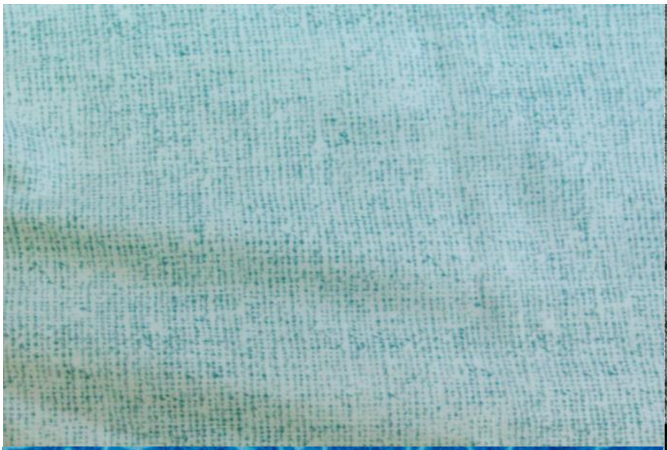

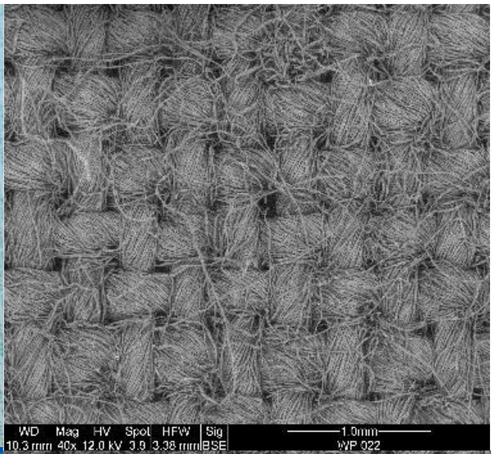

WP023
Mass Market
Quilting
Cotton

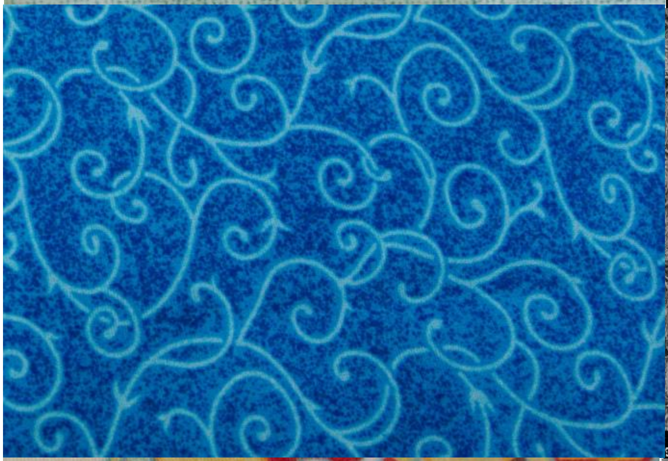

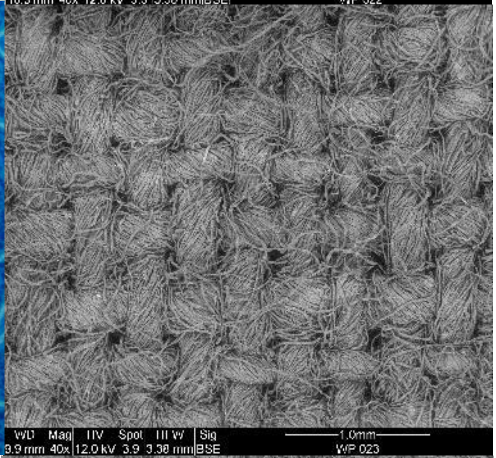

WP024
Home Deco
Fabric

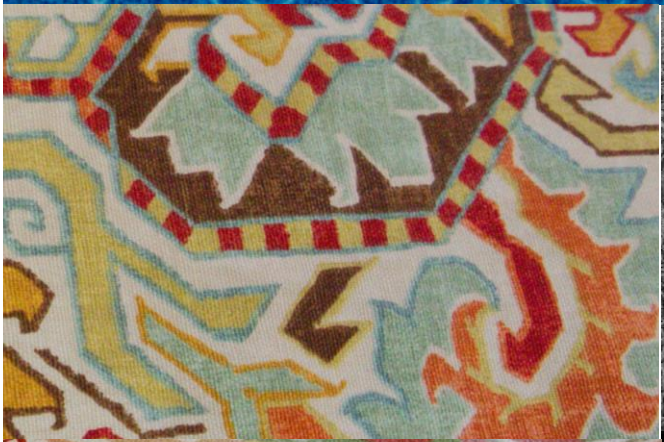

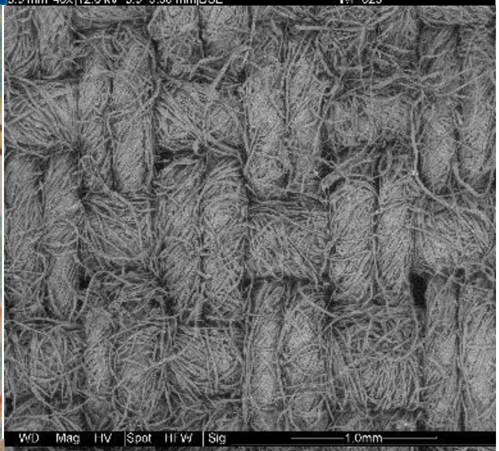

WP025
Home Deco
Fabric

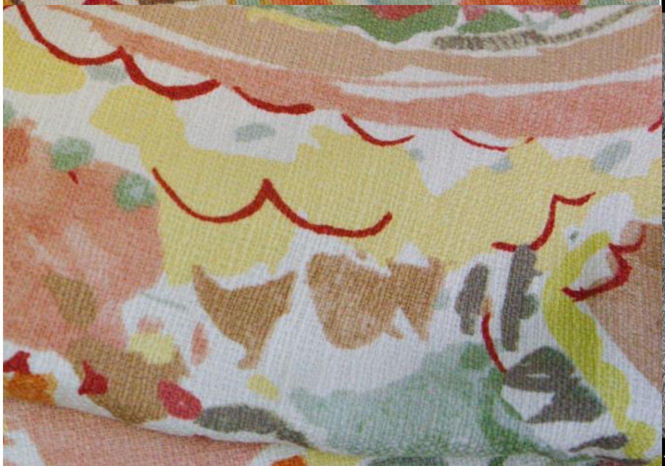

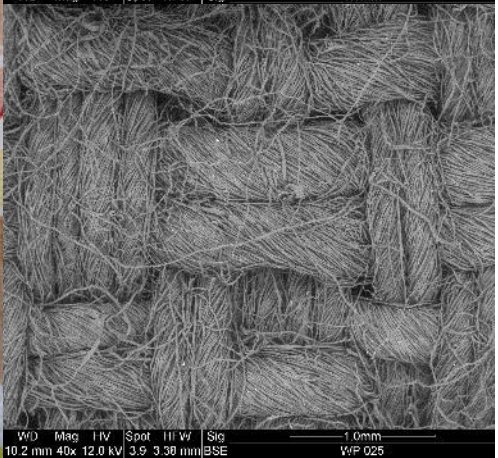

Supplementary Information Fabric Photos and ESEM Images

WP026
Home Deco
Fabric

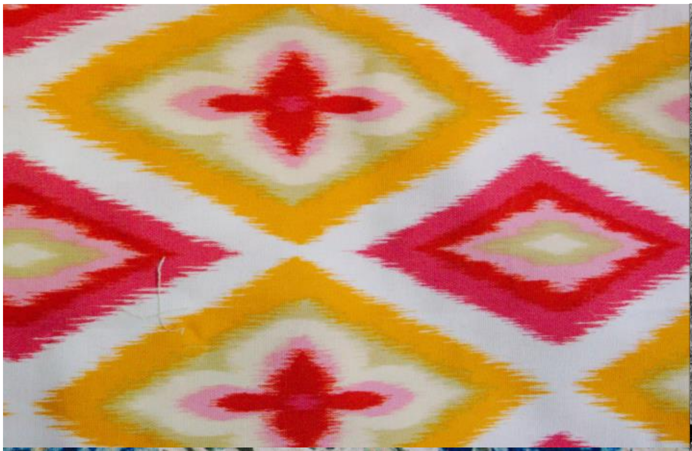

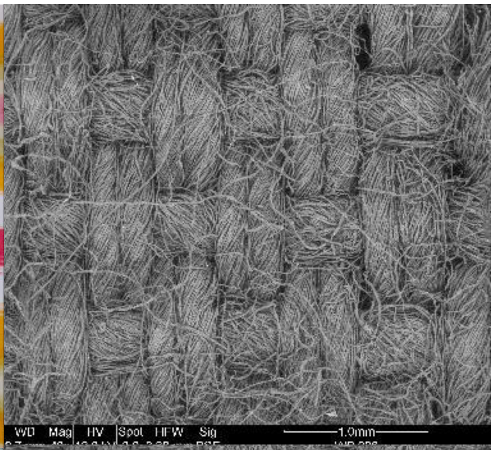

WP027
Home Deco
Fabric

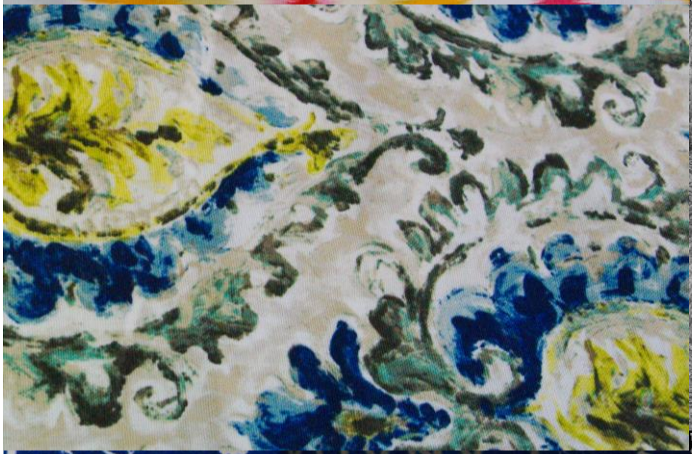

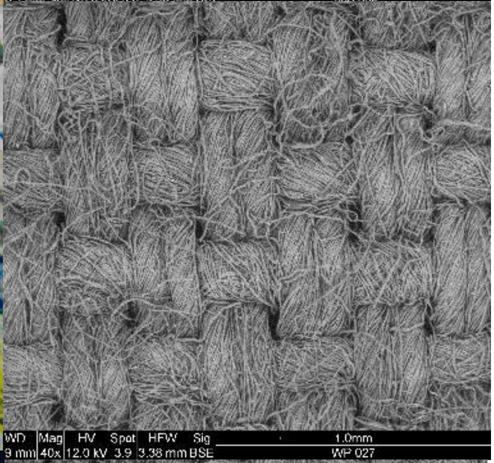

WP052
Home Deco
Fabric

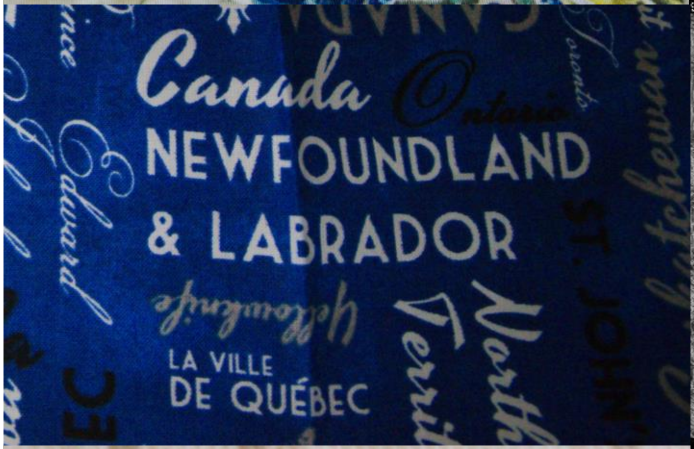

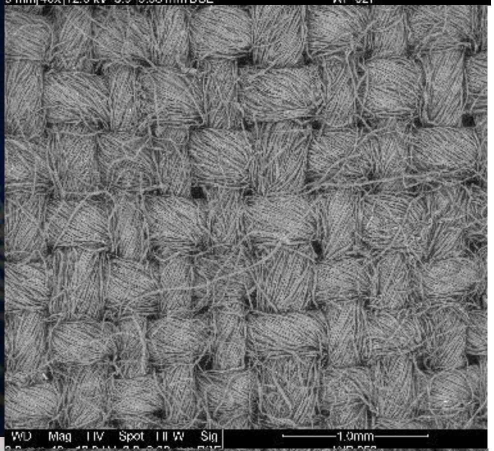

WP028
t-towel

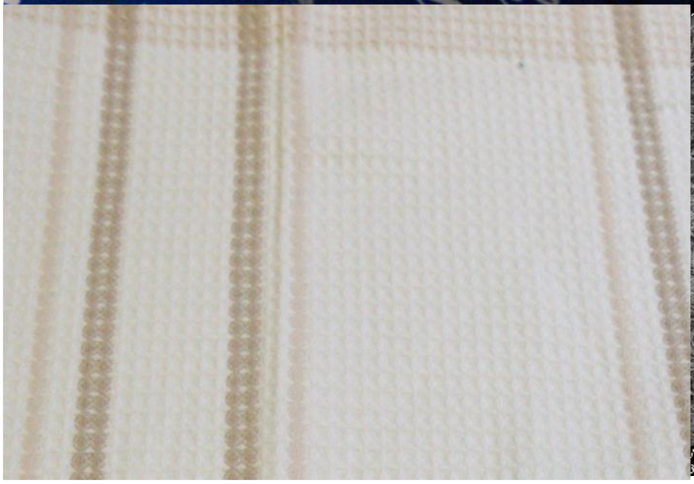

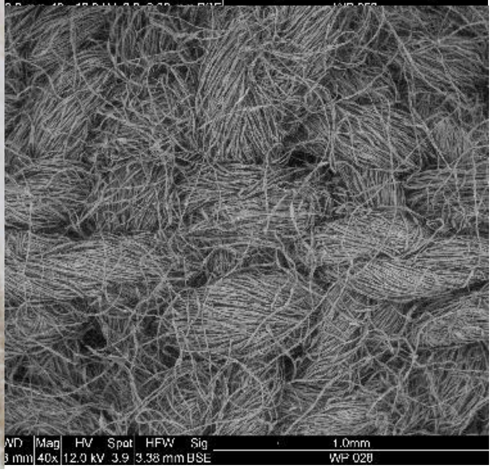

Supplementary Information Fabric Photos and ESEM Images

WP029
t-towel

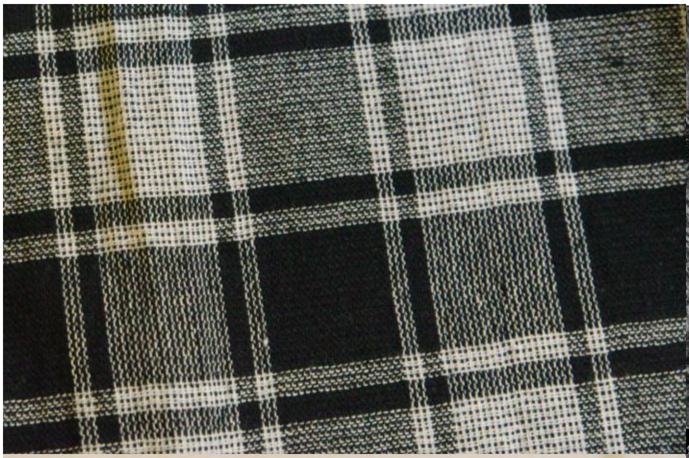

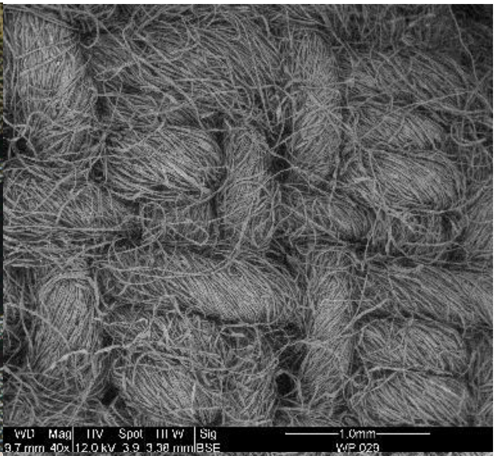

WP030
t-towel

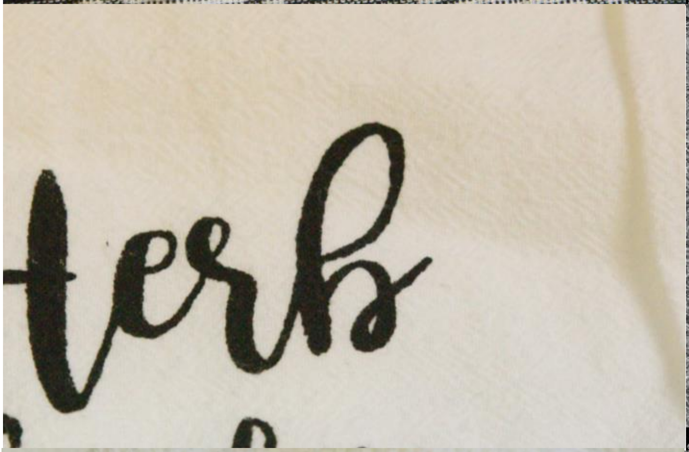

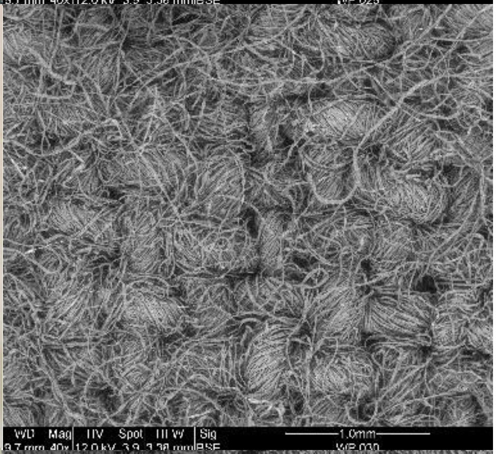

WP031
t-towel

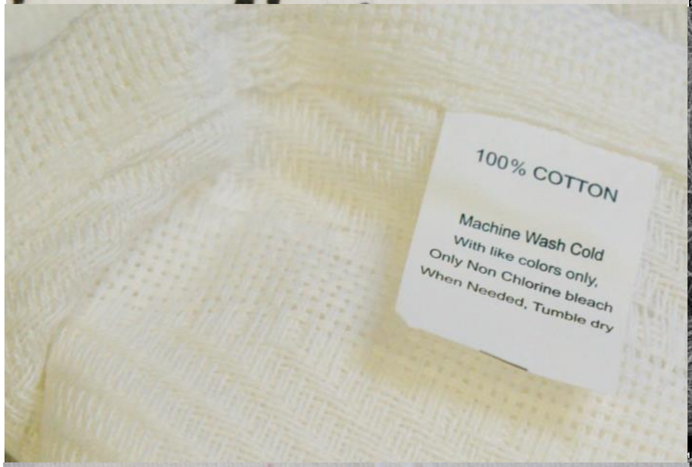

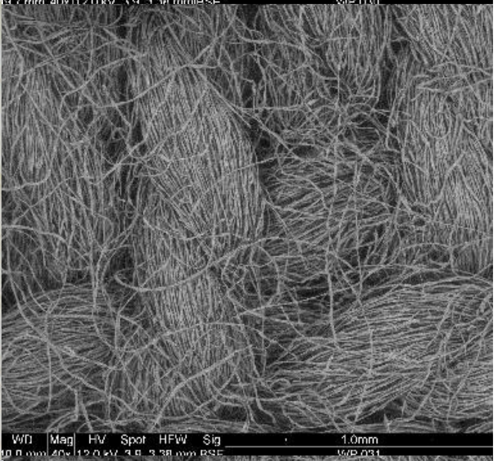

WP032
t-towel

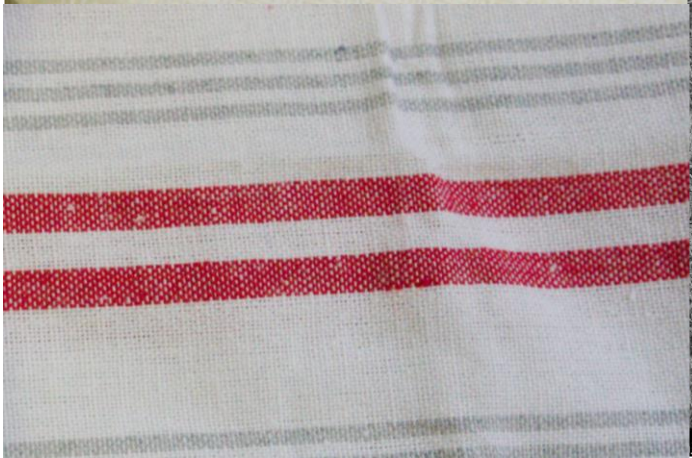

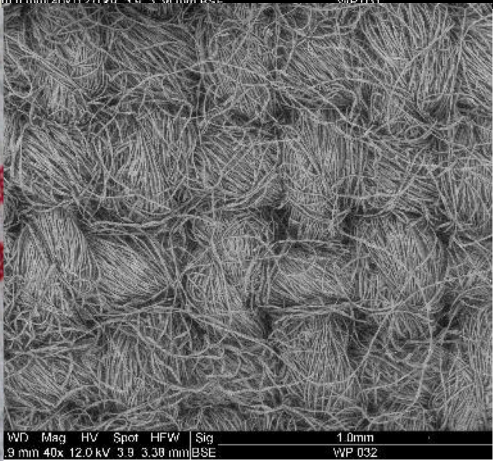

Supplementary Information Fabric Photos and ESEM Images

WP033
t-towel

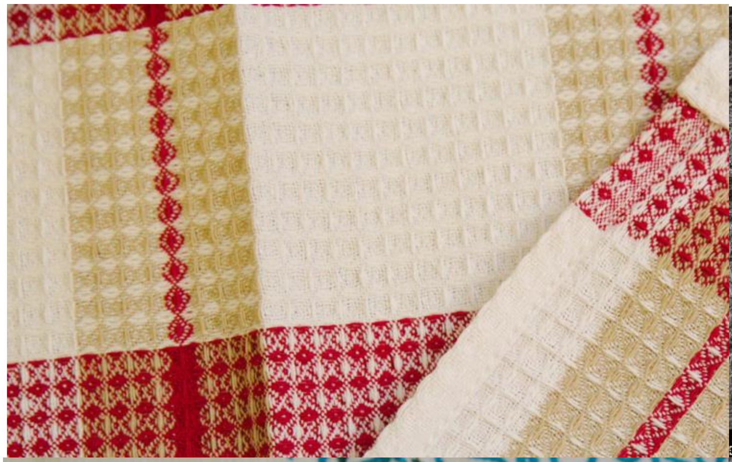

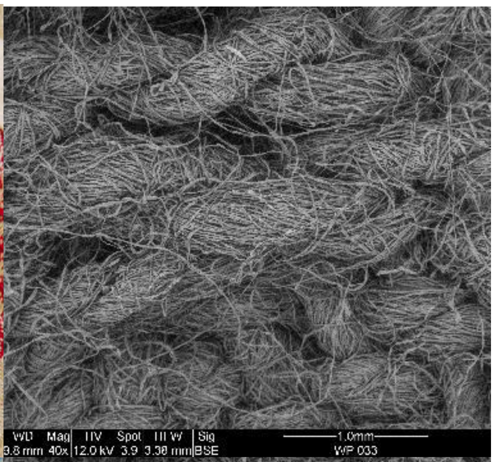

WP034
t-towel

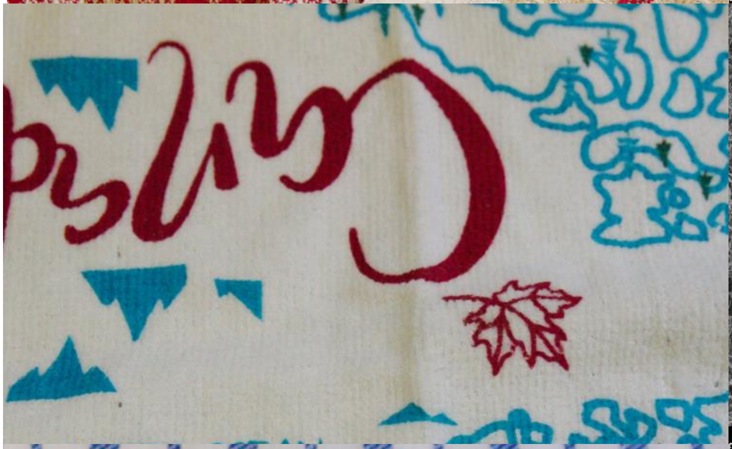

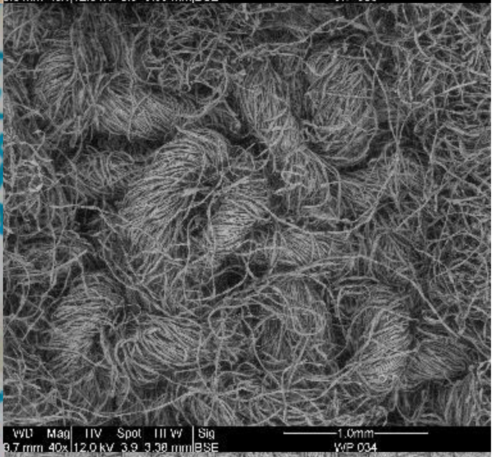

WP035
Bed Sheet

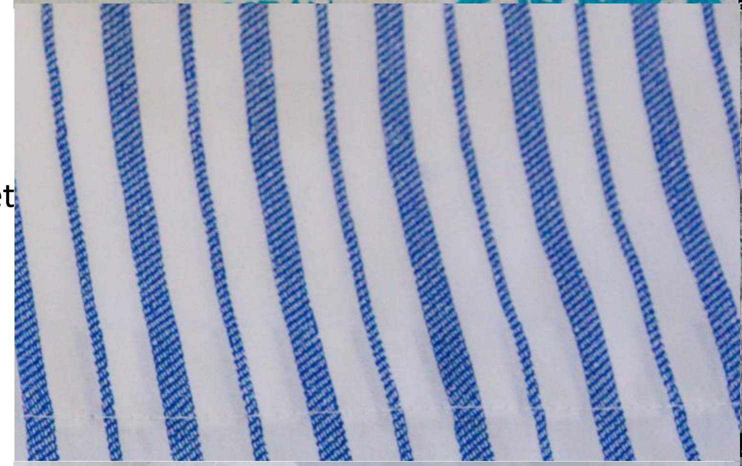

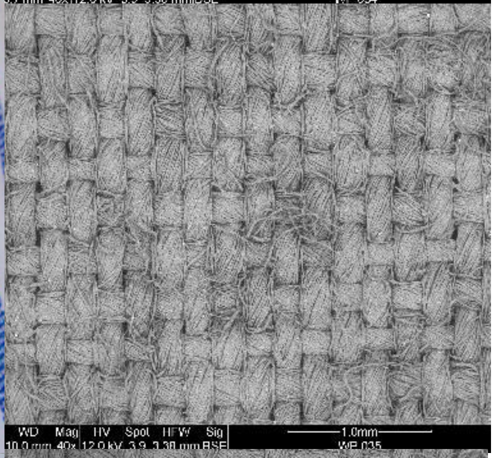

WP036
Bed Sheet

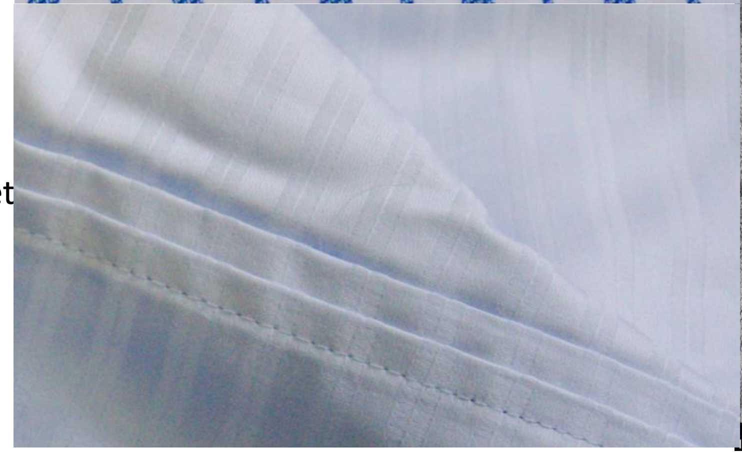

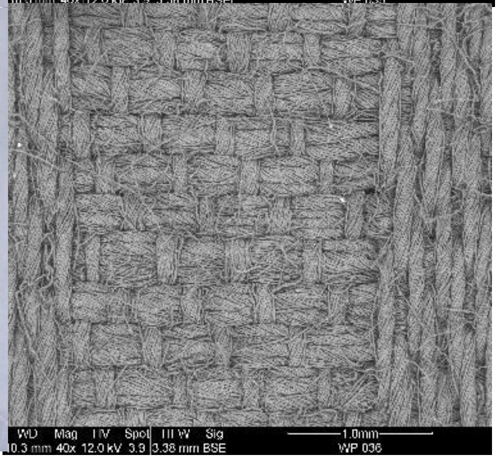

Supplementary Information Fabric Photos and ESEM Images

WP037
Bed Sheet

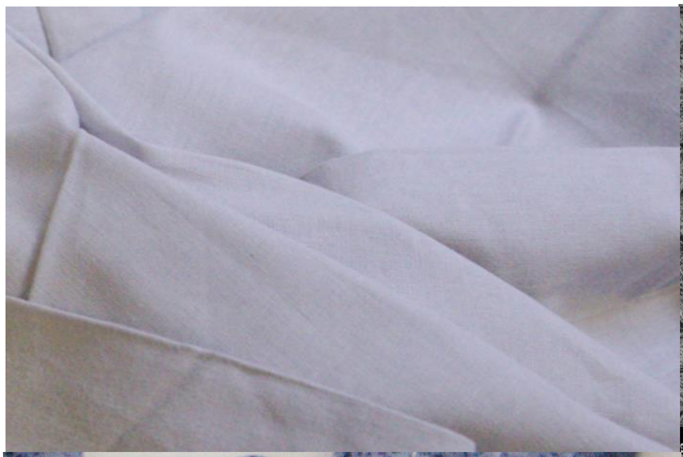

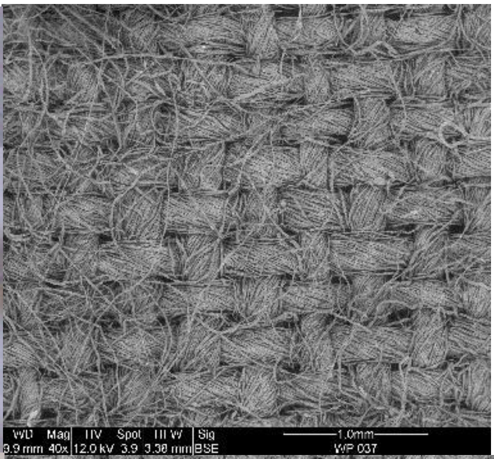

WP038
Bed Sheet

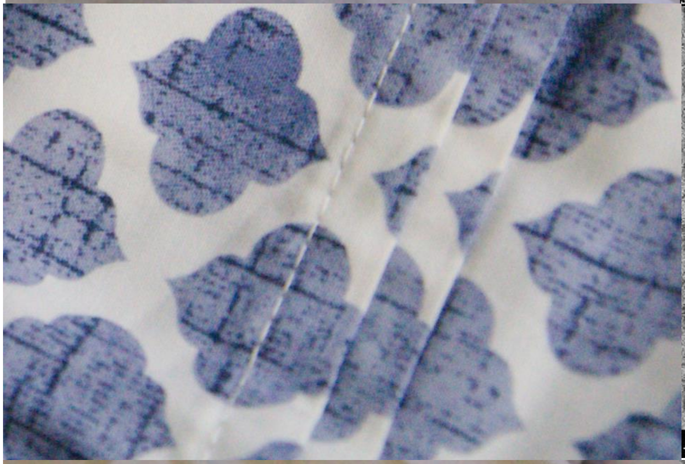

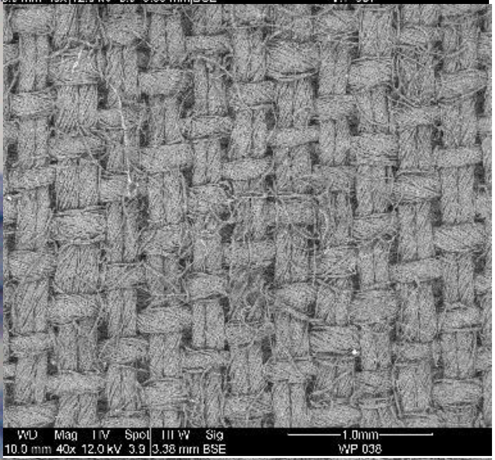

WP039
Bed Sheet

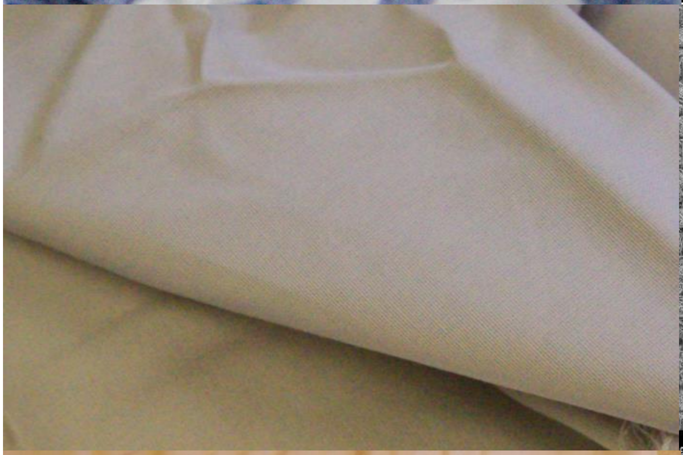

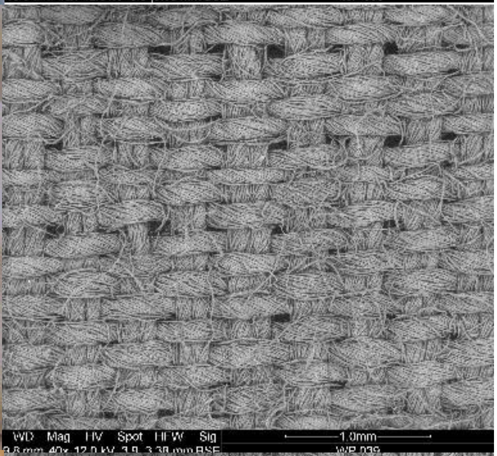

WP040
Bed Sheet

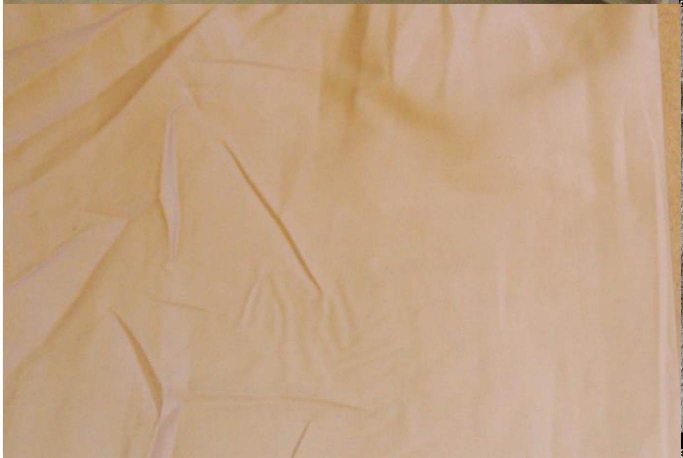

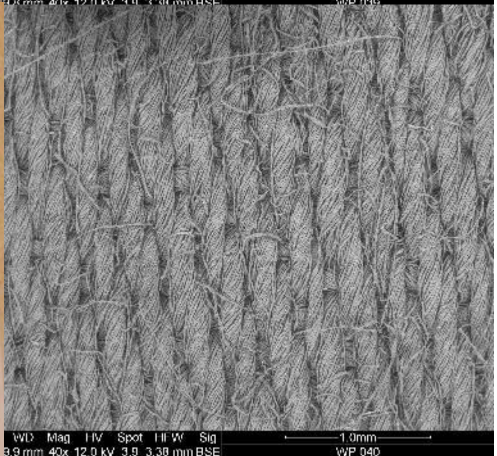

Supplementary Information Fabric Photos and ESEM Images

WP041
High Quality
Quilting
Fabric

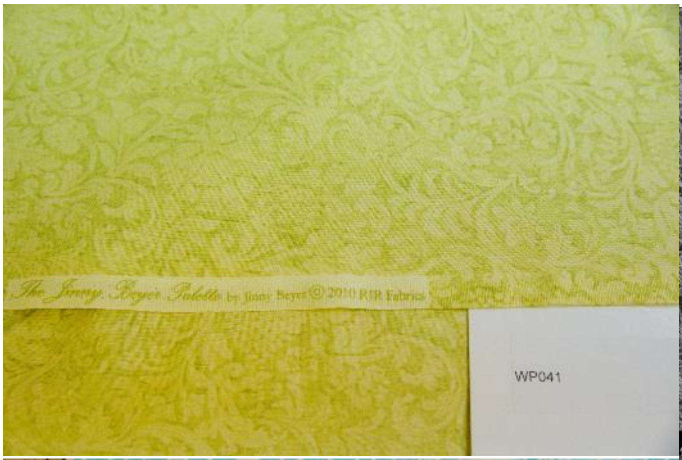

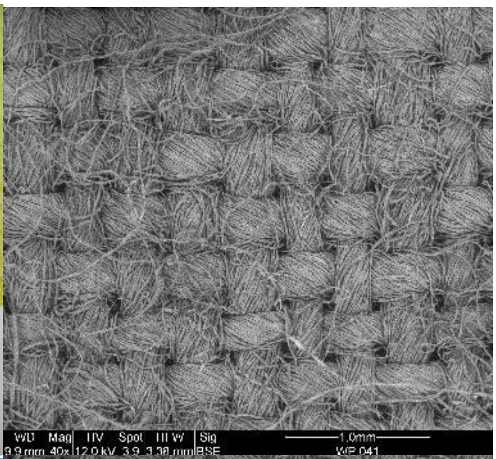

WP042
High Quality
Quilting
Fabric

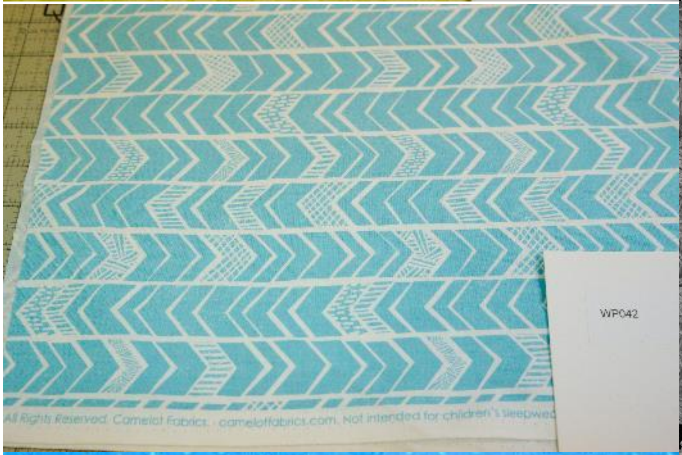

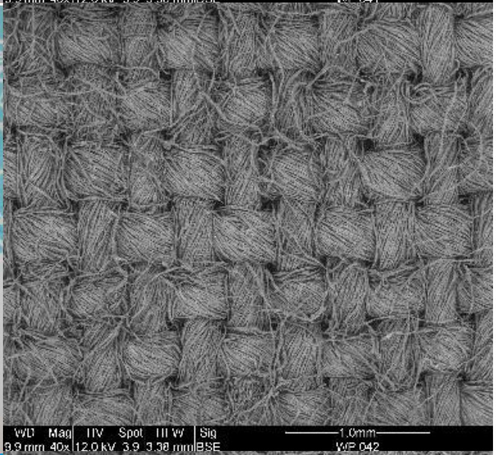

WP043
High Quality
Quilting
Fabric

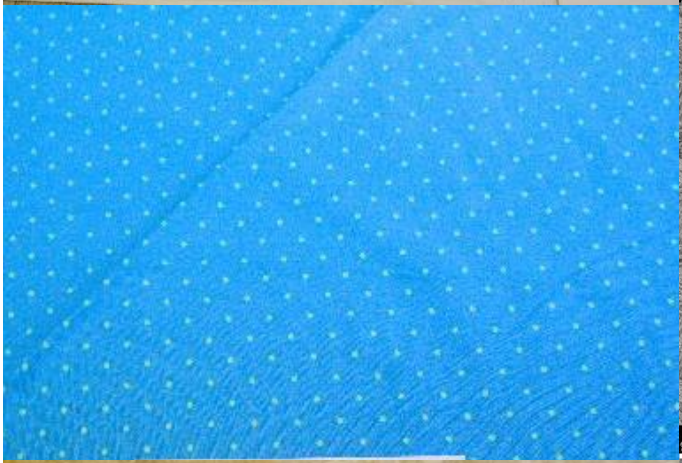

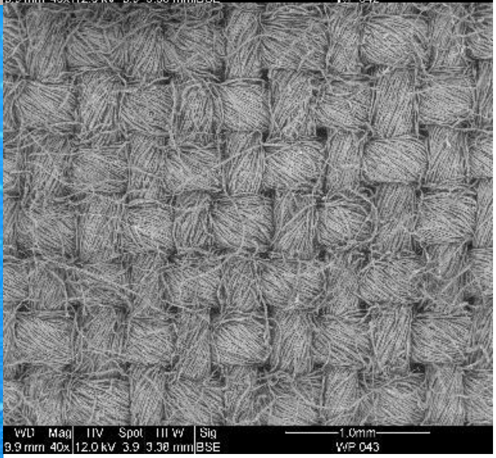

WP044
High Quality
Quilting
Fabric

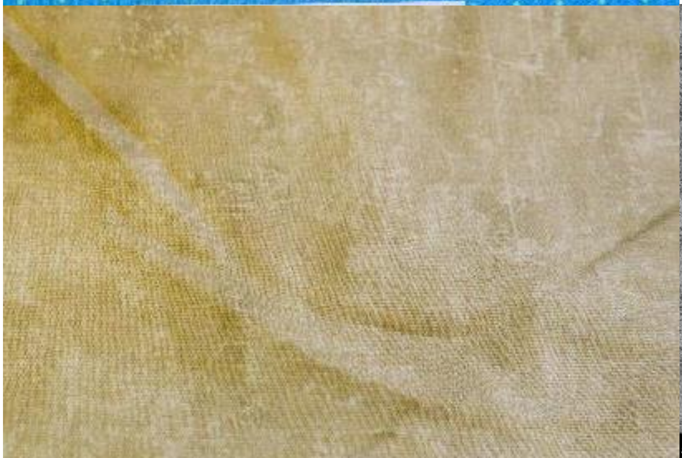

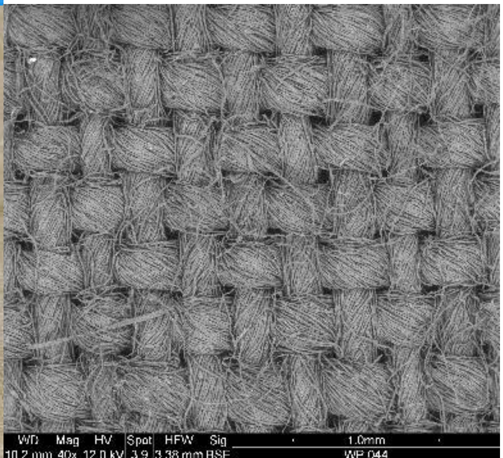

Supplementary Information Fabric Photos and ESEM Images

WP045
High Quality
Quilting
Fabric

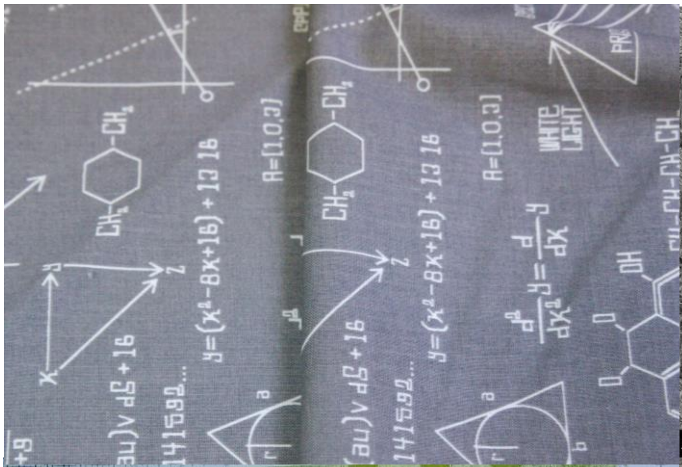

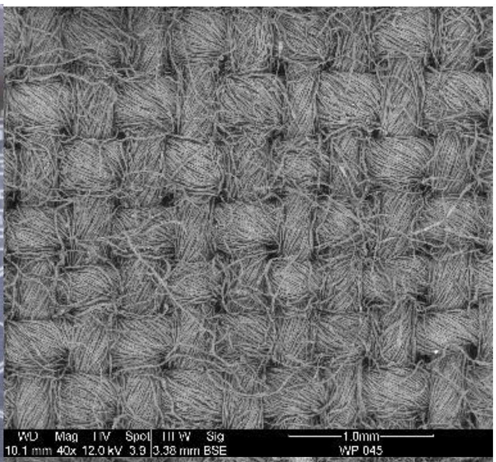

WP046
High Quality
Quilting
Fabric

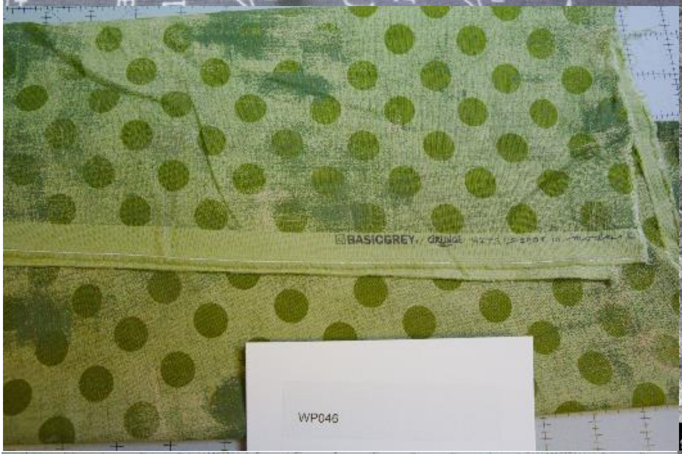

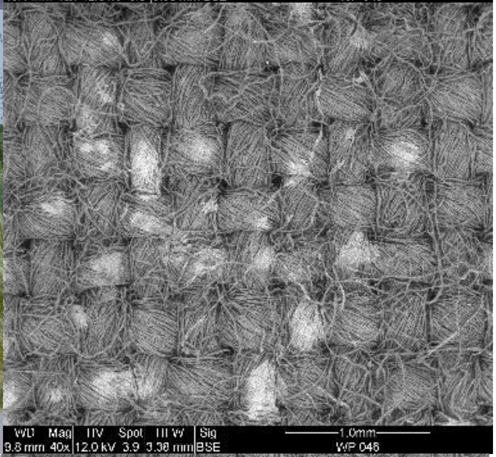

WP047
High Quality
Batic Fabric

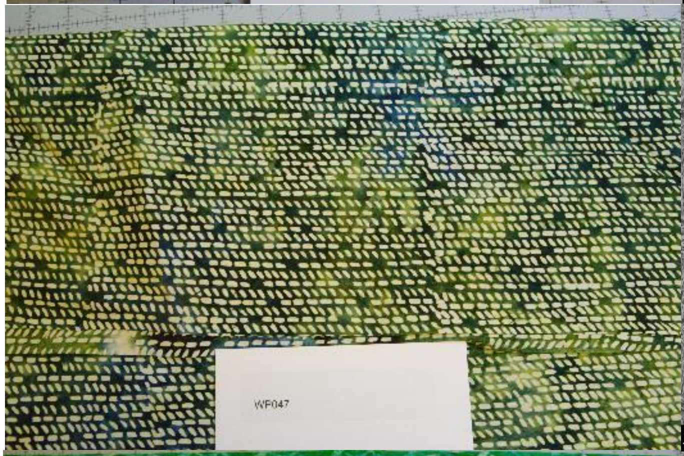

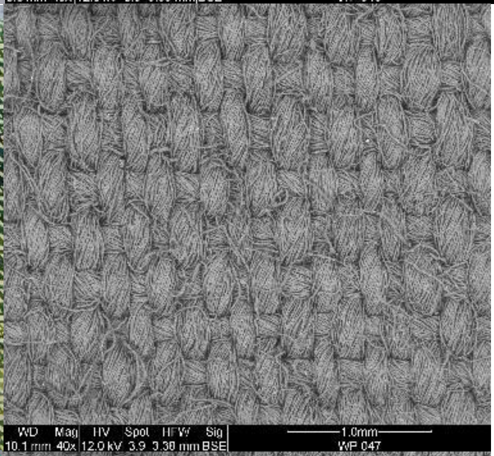

WP048
High Quality
Batik Fabric

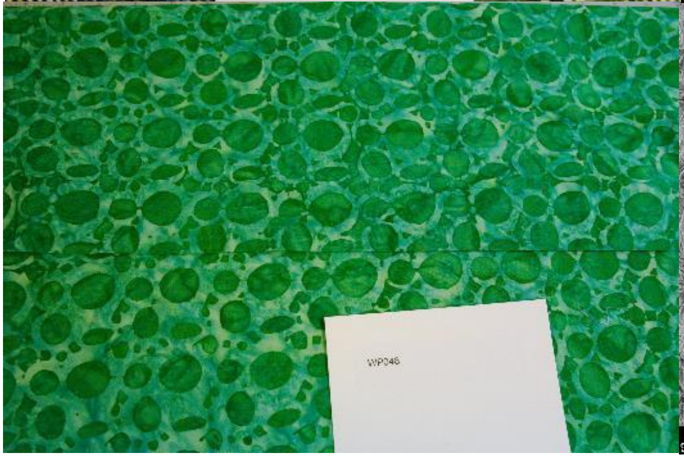

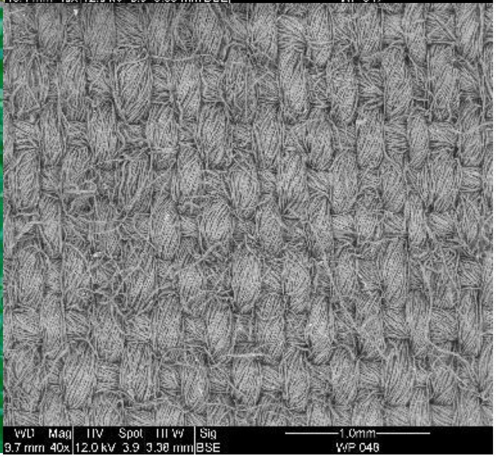

Supplementary Information Fabric Photos and ESEM Images

WP049
High Quality
Batik Fabric

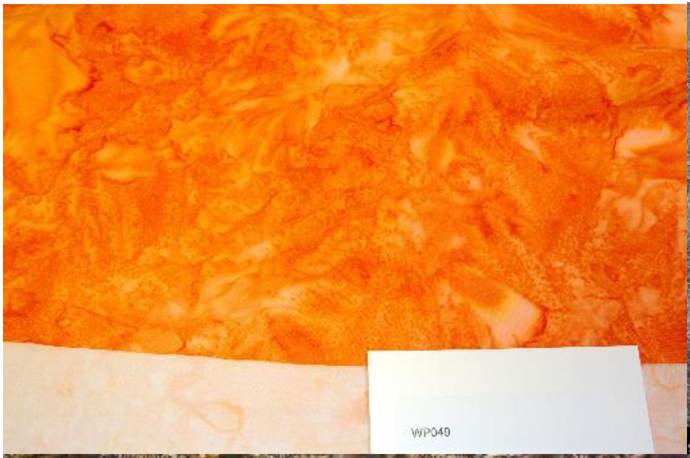

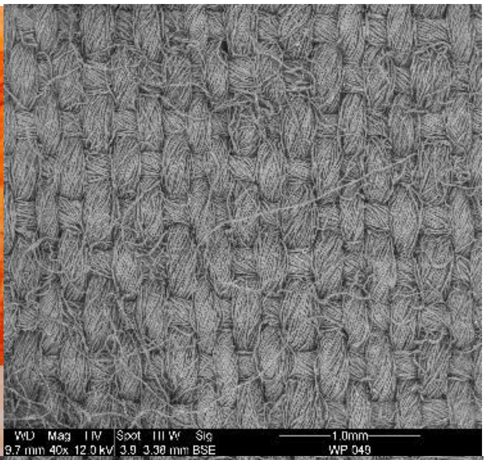

WP050
High Quality
Batik Fabric

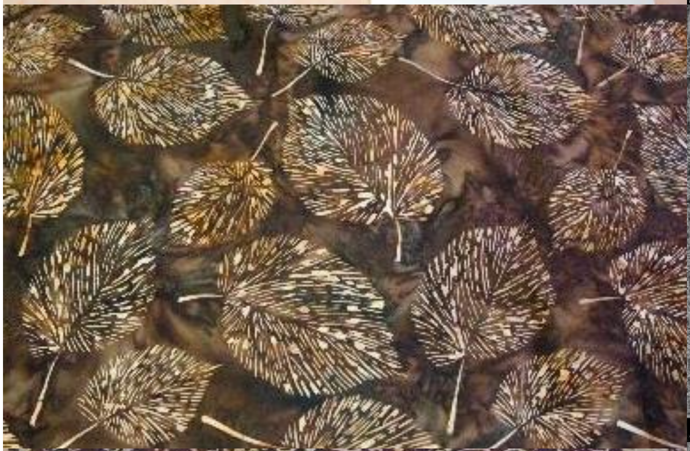

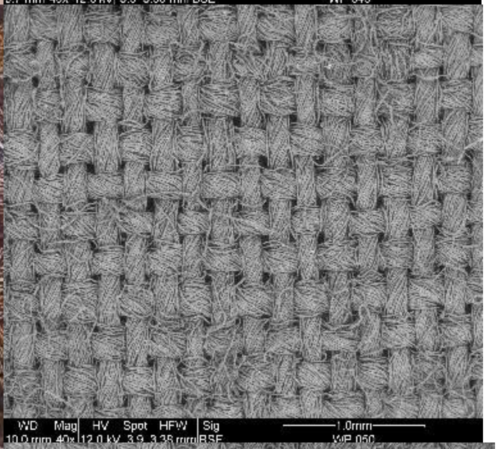

WP051
High Quality
Batik Fabric

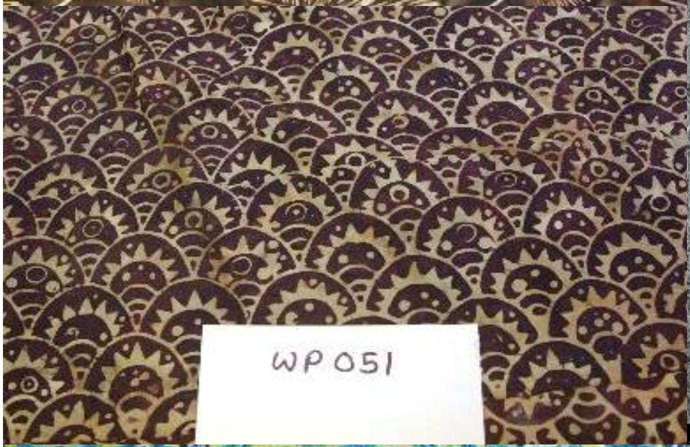

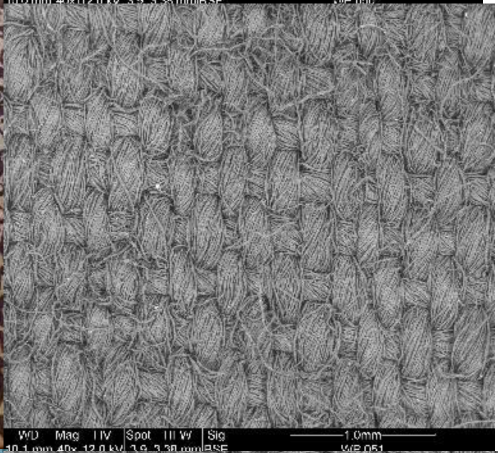

WP053
High Quality
Quilting
Fabric

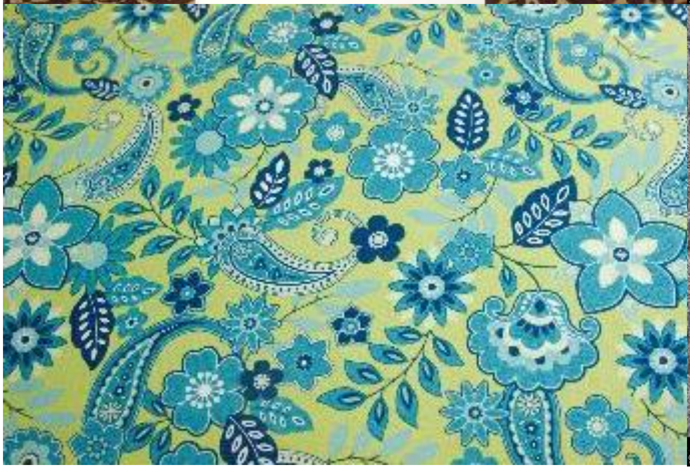

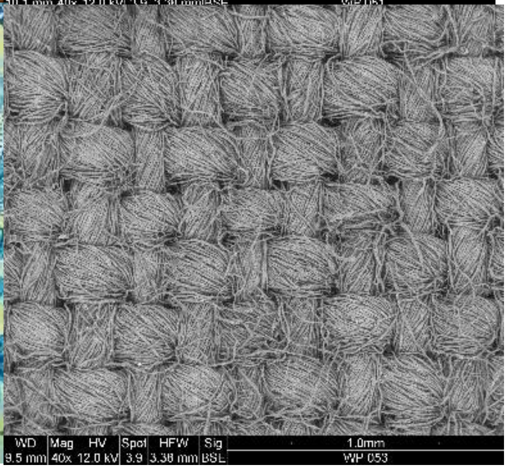

Supplement: S2 File — (PDF) [file pone.0264090.s003.pdf]
